# Supplementary material for: BharatSim: An agent-based modelling framework for India
Source: PLoS Comput Biol. 2024 Dec 30;20(12):e1012682. doi: 10.1371/journal.pcbi.1012682 (PMC11750085; doi:10.1371/journal.pcbi.1012682)
Supplement: S1 Appendix — The simulation times for different population sizes are compared, both for a simple SIR model and the more complicated INDSCI-SIM model described here. We show that the BharatSim simulation engine can handle populations of close to 10 million agents, and that the simulation time scales up as O(N). (PDF) [file pcbi.1012682.s001.pdf]

S1 Appendix: Scaling BharatSim with population size and model complexity

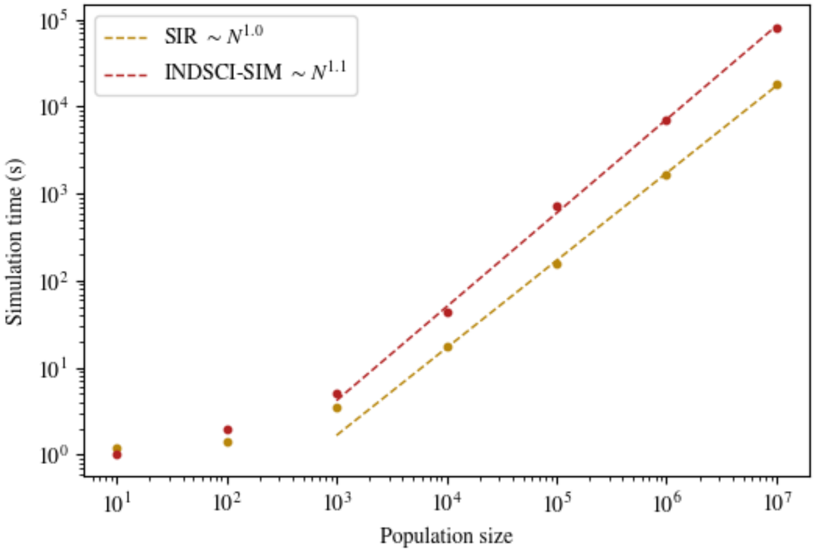

Fig S1.1: Scaling up BharatSim. The times taken to simulate two different models are plotted as a function of population size. In the first, we consider a simple SIR model, and in the second we consider the more complex 9-compartment INDSCI-SIM model that this paper is based on. These times are found to scale roughly as $\mathcal{O}(N)$, with the INDSCI-SIM model being slightly steeper due to the increased model complexity.
