# Supplementary material for: BharatSim: An agent-based modelling framework for India
Source: PLoS Comput Biol. 2024 Dec 30;20(12):e1012682. doi: 10.1371/journal.pcbi.1012682 (PMC11750085; doi:10.1371/journal.pcbi.1012682)
Supplement: S2 Appendix — We benchmark the quality of the synthetic population by comparing the statistics of the numerical and categorical data in both the synthetic population and survey data. In each case, a similarity measure is reported. (PDF) [file pcbi.1012682.s002.pdf]

S2 Appendix: Creating and benchmarking a synthetic population for Mumbai city

2.1 Generating the Mumbai synthetic population

Located in the state of Maharashtra in India, Mumbai is one of India's largest cities, with a population of over 12 million. We use a subset of the IHDS-II dataset obtained by filtering for individuals and households which are situated in the state of Maharashtra. (We use the filtered datasets for Maharashtra because the Mumbai dataset in IHDS-II contains a few hundred samples, which are not enough to be able to generate a quality synthetic population.) Each job description is drawn from the empirical distribution observed in the IHDS-II subset for Maharashtra.

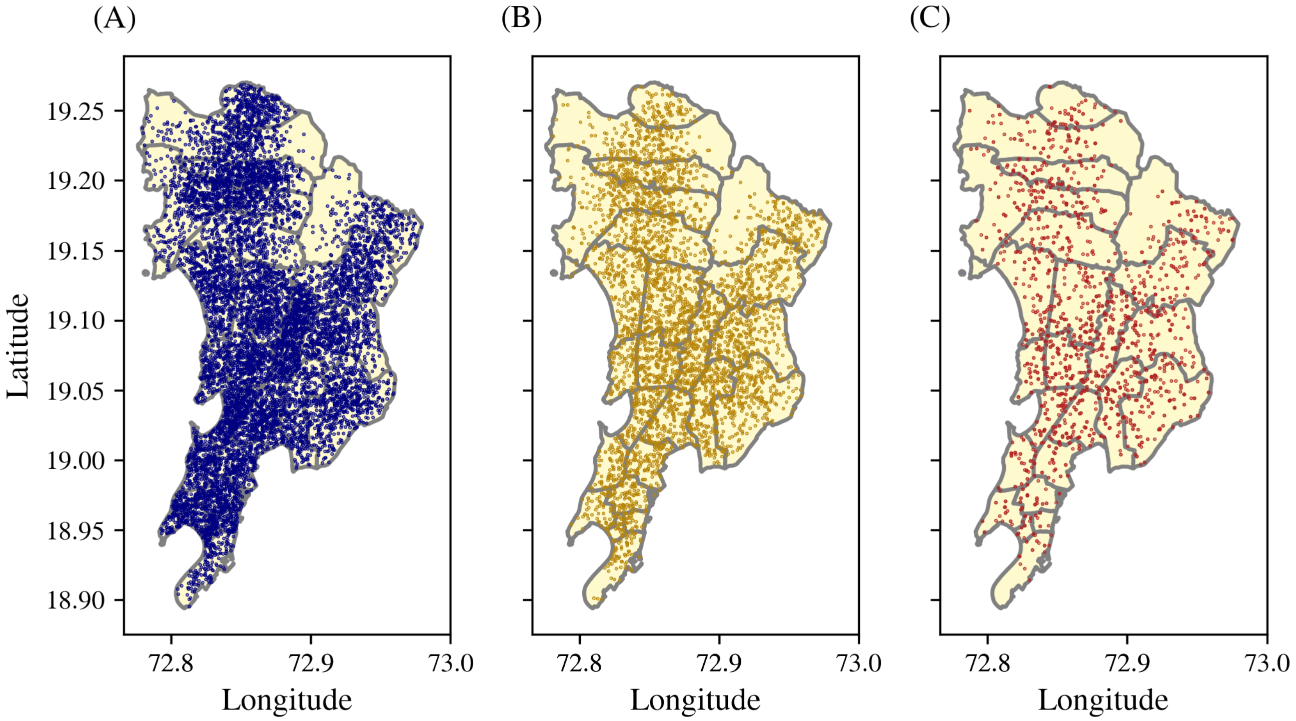

Fig S2.1: Distribution of geo-locations. Geographical distribution of (A) households, (B) workplaces, and (C) schools for the combined synthetic population of the districts of Mumbai and Mumbai Suburban. The underlying map of Mumbai is provided by the Spatial Data of Municipalities (Maps) Project by Data{Meet} [1].

Next, we generate synthetic workplaces, schools, and public places for our agents as detailed in Section 2.1.2 in the main paper. The final synthetic population has demographic data (age, height, and so on), disease data (chronic heart disease, diabetes, and other co-morbidities), family data, geographic location for workplaces, schools, and other geo-locations, and socio-economic data (job label, and religion). In total, we generate a synthetic population with 44 attributes. These attributes and their datatypes are shown in Table S2.1.

Col	Title	Datatype	Col	Title	Datatype
1	AgentID	int64	23	District	string
2	SexLabel	string	24	JobType	string
3	Age	int64	25	EssentialWorker	bool
4	Height	float64	26	AdminUnit_Name	string
5	Weight	float64	27	AdminUnit_Lat	float64
6	Religion	string	28	AdminUnit_Lon	float64
7	Caste	string	29	HHID	int64
8	M_Fever	bool	30	H_Lat	float64
9	M_Cough	bool	31	H_Lon	float64
10	M_Diarrhea	bool	32	AdherenceToIntervention	float64
11	M_Cataract	bool	33	UsesPublicTransport	bool
12	M_TB	bool	34	WorkPlaceID	int64
13	M_HighBP	bool	35	W_Lat	float64
14	M_HeartDisease	bool	36	W_Lon	float64
15	M_Diabetes	bool	38	WorkPlace_AdminUnit	string
16	M_Leprosy	bool	38	SchoolID	int64
17	M_Cancer	bool	39	School_Lat	float64
18	M_Asthma	bool	40	School_Lon	float64
19	M_Polio	bool	41	School_AdminUnit	string
20	M_Paralysis	bool	42	PublicPlaceID	int64
21	M_Epilepsy	bool	43	PublicPlace_Lat	float64
22	StateLabel	string	44	PublicPlace_Lon	float64

Table S2.1: Columns in the synthetic population. The 44 attributes present in the created synthetic population and their datatypes are shown.

2.2 Benchmarking our population against survey data

Fig S2.2 compares the marginal distributions of attributes of age, height, and weight across samples of the survey and the synthetic population for the combined districts of Mumbai and Mumbai Suburban. We work with a randomly chosen subset comprising 10,000 individuals to compare with the survey data, although our synthetic population has 12 million individuals.

Table S2.2 shows results for two statistical tests, the two-sample Kolmogorov–Smirnov (KS) test and the Chi-Squared (CS) test. These statistical tests are conducted on all the compatible columns in both the survey and synthetic population, so that the CS test is applied to categorical or Boolean columns, and the KS test is applied to numerical columns. In each case, we report a confidence level for each test that represents the confidence that the synthetic data and survey data come from the same distribution. For the CS test, the confidence level is the $p-$value, while for the KS test it is 1 - (KS statistic). The average confidence levels for relevant columns are reported in Table S2.2.

2.3 Metrics for the synthetic population

To further study how faithfully our synthetic population represents the survey data from the IHDS-II dataset, we run a series of tests to compare their statistical features. The data in our population is of two types: numerical and categorical. We use different tests to check the similarity between

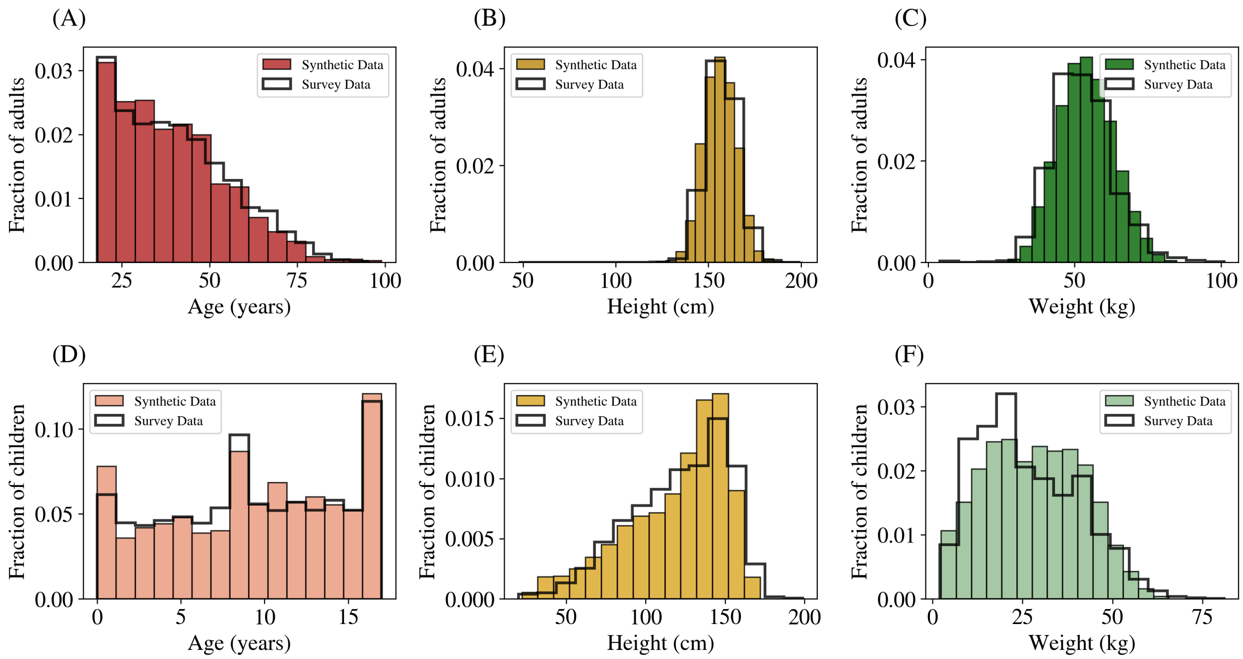

Fig S2.2: Histograms of marginal distributions. The distributions of age, height, and weight in the synthetic population for the combined districts of Mumbai and Mumbai Suburban. (A), (B), and (C) show this distributions for adults, while (D), (E), and (F) show the same distributions for children. In each case, this distribution is plotted over the distributions obtained from survey data, and these distributions compared using the Kolmogorov-Smirnov test. The results and KS statistic for each case are given in Table S2.2.

Test	Confidence level
CS Test for Sex	0.99
KS Test for Age	0.98
KS Test for Height	0.91
KS Test for Weight	0.95

Table S2.2: Statistical benchmarks of the synthetic population. To compare the distributions of various columns, we use the chi-square test for columns with discrete, categorical data (sex in our population). This test returns a $p-$value. A high $p-$value indicates the confidence that the synthetic data and the survey data come from the same discrete distribution. We also use the Kolmogorov-Smirnov statistic to compare the marginal distributions of continuous, numerical data (age, height, and weight in our population). We report a score of 1-(KS statistic). A higher score again indicates the confidence that the synthetic data and the survey data come from the same continuous distribution.

the generated population and the survey dataset. Three of these tests are for categorical columns: `CategoryCoverage`, `ContingencySimilarity`, and `TVComplement`, while the others are for the numerical columns. We report the results for the tests described below on columns that are present in both the generated synthetic population and the IHDS dataset. These results are shown in Tables S2.3 and S2.4. In every test a result of 1.0 signifies that the survey and synthetic population

are identical, and 0.0 signifies that they survey and synthetic population are very dissimilar.

`CategoryCoverage` Checks whether the synthetic column covers all the categories present in the survey column.

`TVComplement` Computes the similarity between synthetic and survey categorical columns in terms of their marginal distributions.

`BoundaryAdherence` Checks whether a column from the synthetic data respects the range of the survey data or not. It does so by comparing the minimum and maximum values from both datasets.

`CorrelationSimilarity` Compares the trends of 2D distributions by measuring the Pearson correlation coefficient between a pair of numerical columns and computing the similarity between the survey and synthetic datasets.

`RangeCoverage` Checks whether the synthetic column covers the entire range of values present in the survey column.

`KSComplement` Computes the similarity between synthetic and survey numerical columns in terms of their marginal distributions.

`ContingencySimilarity` Compares the 2D distributions by computing the similarity of a pair of columns between the survey and synthetic categorical columns.

`StatisticSimilarity` Using mean, median and standard deviation it computes the similarity between a survey and synthetic numerical column.

We also consider joint tests for numerical columns to verify that correlations between the different columns are faithfully represented.

References

[1] Data{Meet} Community. Spatial data of Municipalities; 2024. Available from: http://projects.datameet.org/Municipal_Spatial_Data/.

Features	BoundaryAdherence	RangeCoverage	StatisticSimilarity	KSComplement
Height	0.9999	0.9879	0.9893	0.9474
Weight	1.0000	0.8400	0.9657	0.8907
Age	0.9990	1.0000	0.9851	0.9176

Feature	TVComplement
SexLabel	0.9296
M_Cough	0.9996
M_Cancer	0.9997
M_Diarrhea	0.9995
M_Fever	0.9997
M_Cataract	0.9992
M_TB	1.0000
M_HeartDisease	0.9987
M_Diabetes	0.9999
M_HighBP	0.9997
M_Leprosy	0.9998
M_Asthma	0.9996
M_Paralysis	0.9996
M_Epilepsy	0.9981
M_Polio	1.0000

Table S2.3: Metrics for comparing numerical (age, height, and weight) and categorical (comorbidity) columns between the Mumbai synthetic population and the IHDS-II survey data. In every test a result of 1.0 signifies strong correlation and 0.0 signifies no correlation between the survey and synthetic data.

Features	CorrelationSimilarity
Age, Height	0.9999
Age, Weight	0.9838
Height, Weight	0.9754

Features	ContingencySimilarity
M_Cough, M_HeartDisease	0.9983
M_Diabetes, M_HeartDisease	0.9987
M_Cough, M_Fever	0.9994
M_Cough, M_Asthma	0.9994

Table S2.4: Metrics for comparing the joint distributions of numerical (age, height, and weight) and categorical (comorbidity) columns between the Mumbai synthetic population and survey data from the IHDS-II survey. In every test a result of 1.0 signifies strong correlation and 0.0 signifies no correlation between the survey and synthetic data.
