# Supplementary material for: BharatSim: An agent-based modelling framework for India
Source: PLoS Comput Biol. 2024 Dec 30;20(12):e1012682. doi: 10.1371/journal.pcbi.1012682 (PMC11750085; doi:10.1371/journal.pcbi.1012682)
Supplement: S3 Appendix — The disease-progression in a single well-mixed compartmental model is briefly described, and an association is made between the results of this compartmental model and a well-mixed (all-to-all connected) agent-based model with the same compartments. (PDF) [file pcbi.1012682.s003.pdf]

S3 Appendix: Description of the compartmental model

As mentioned in the main text, the INDSCI-SIM model contains 9-compartments. These are (S)usceptible, (E)xposed, Asymptomatic (I^A), Presymptomatic (I^P), Mildly Infected (I^M), Severely Infected (I^S), (H)ospitalised, (R)ecovered, and (D)ead. These are shown in Fig 5 of the main text. Furthermore, each of these compartments is age-stratified, as denoted by the subscript i. Using these definitions, the dynamics of our compartmental model can be represented by the following equations:

$$\begin{aligned}
\dot{S}_i &= -\beta S_i \sum_{j=1}^{N_{\text{age}}} I_j^{\text{eff}} + \zeta R_i \\
\dot{E}_i &= \beta S_i \sum_{j=1}^{N_{\text{age}}} I_j^{\text{eff}} - \gamma E_i \\
\dot{I}_i^A &= \alpha \gamma E_i - \lambda_A I_i^A \\
\dot{I}_i^P &= (1-\alpha)\gamma E_i - \lambda_P I_i^P \\
\dot{I}_i^M &= \mu \lambda_P I_i^P - \lambda_M I_i^M \\
\dot{I}_i^S &= (1-\mu)\lambda_P I_i^P - \lambda_S I_i^S \\
\dot{H}_i &= \lambda_S I_i^S - \rho H_i \\
\dot{R}_i &= (1-\delta)\rho H_i + \lambda_A I_i^A + \lambda_M I_i^M - \zeta R_i \\
\dot{D}_i &= \delta \rho H_i
\end{aligned} \tag{1}$$

where the parameters are described in Table S3.1, and I_j^{eff} is given by:

$$I_j^{\text{eff}} = \left(C_A I_j^A + C_P I_j^P + C_M I_j^M + C_S I_j^S \right) / N_j .$$

Parameter	Description
β	Rate at which infected individuals can infect the susceptible population
γ	Rate at which exposed individuals become infectious
λ_A	Asymptomatic individuals remain infected for an average of $1/\lambda_A$ days
λ_P	Presymptomatic individuals remain infected for an average of $1/\lambda_P$ days
λ_M	Individuals experiencing mild symptoms recover on an average in $1/\lambda_M$ days
λ_S	Individuals experiencing severe symptoms are hospitalised on an average in $1/\lambda_S$ days
ρ	Hospitalised individuals remain hospitalised for an average of $1/\rho$ days
ζ	Recovered individuals remain immune to the disease for an average of $1/\zeta$ days
α	Fraction of exposed individuals who are asymptomatic carriers of the disease
μ	Fraction of presymptomatic carriers who become mildly infected
δ	Fraction of hospitalised individuals who die from the disease
C_A	Contact parameter: relative risk of an asymptomatic agent infecting a susceptible agent
C_P	Contact parameter: relative risk of a presymptomatic agent infecting a susceptible agent
C_M	Contact parameter: relative risk of a mildly-infected agent infecting a susceptible agent
C_S	Contact parameter: relative risk of a severely-infected agent infecting a susceptible agent

Table S3.1: Description of the parameters used in Eq S1.

However, it is important to stress that we only use the compartments described in this model to label the different states in the disease progression. The dynamics described in the paper differ significantly from those of the compartmental model above in two significant ways: (i) our model in the paper has multiple network interactions and therefore incorporates differential contacts between agents, both spatially and temporally, and (ii) the sojourn times of agents in the different disease compartments of our model are taken to be lognormally distributed (see Table S3 of the main paper), while those of the well-mixed compartmental model are exponentially distributed.

3.1 On the equivalence of compartmental and well-mixed agent-based model

In this supplementary section, we show that we can closely reproduce results from the above compartmental model using our agent-based simulation. We do this by (i) simulating a well-mixed scenario in which every agent interacts with with each other agent at any given time, and (ii) incorporating exponential, rather than lognormally distributed, residence times.

In Fig S3.1 we show the results for the total number of active infections in an agent-based simulation (averaged over multiple stochastic runs), and compare it to the results obtained by solving the differential equations in Eq S1. These simulations are run using the parameters defined in Table S3.2.

Parameter	Value	Parameter	Value	Parameter	Value
C_A	1.0	γ	1/5	ρ	1/18
C_P	1.0	λ_A	1/5	ζ	0.0
C_M	1.0	λ_P	1/2	α	0.35
C_S	1.0	λ_M	1/8	μ	0.9792
β	0.3	λ_S	1/2	δ	0.00032

Table S3.2: Parameter values used in comparing the agent-based and differential equation model (described by Eq S1).

In Fig S3.1 we show the epidemic curves for all infected individuals in the population, both using our agent-based simulator and using the differential equation based compartmental model described in Eq S1. We see from the figure that while a small difference exists between the results from the compartmental model and those of our agent-based simulations, this difference reduces as the time-step of the agent-based simulation is reduced. This difference, that we attribute to the discrete-time nature of our agent-based simulator, can be accounted for by a small change in the parameter β.

In Fig S3.2, we show the results for all compartments in the disease progression, from which we conclude that our agent-based simulation does indeed agree with the results from the compartmental model. We show further in Fig S3.3 that a perfect matching between the agent-based and compartmental results can be obtained by allowing a single parameter (β) to vary by less than 5% (i.e. changing $\beta = 0.3$ to $\beta = 0.31$).

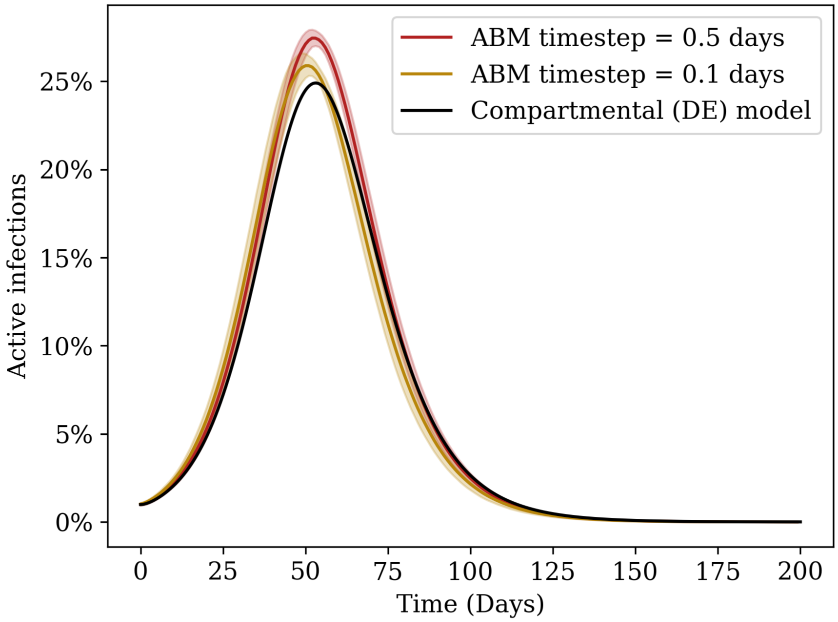

Fig S3.1: Comparing agent-based and differential-equation based models. Epidemic curves showing the total infected individuals in the population as a function of time. The red curve shows the results using a time-step of half-a-day in our agent-based simulations, while the golden curve shows the results using a time-step of one-tenth of a day. In each case, a population of 100,000 well-mixed individuals was considered, and 200 simulations were run and averaged over. The shaded regions represent error bars of 1.96σ. The black solid curve shows the same curve for the compartmental model, obtained by solving the differential equations in Eq S1 with the same initial conditions. While the results of our model differ marginally from those of the compartmental model, we attribute this to the discrete time-step used. Indeed, as the time-step is lowered, a stronger agreement is seen. The models can be made to match exactly if the parameter β is slightly changed, to account for the discrete-time effects.

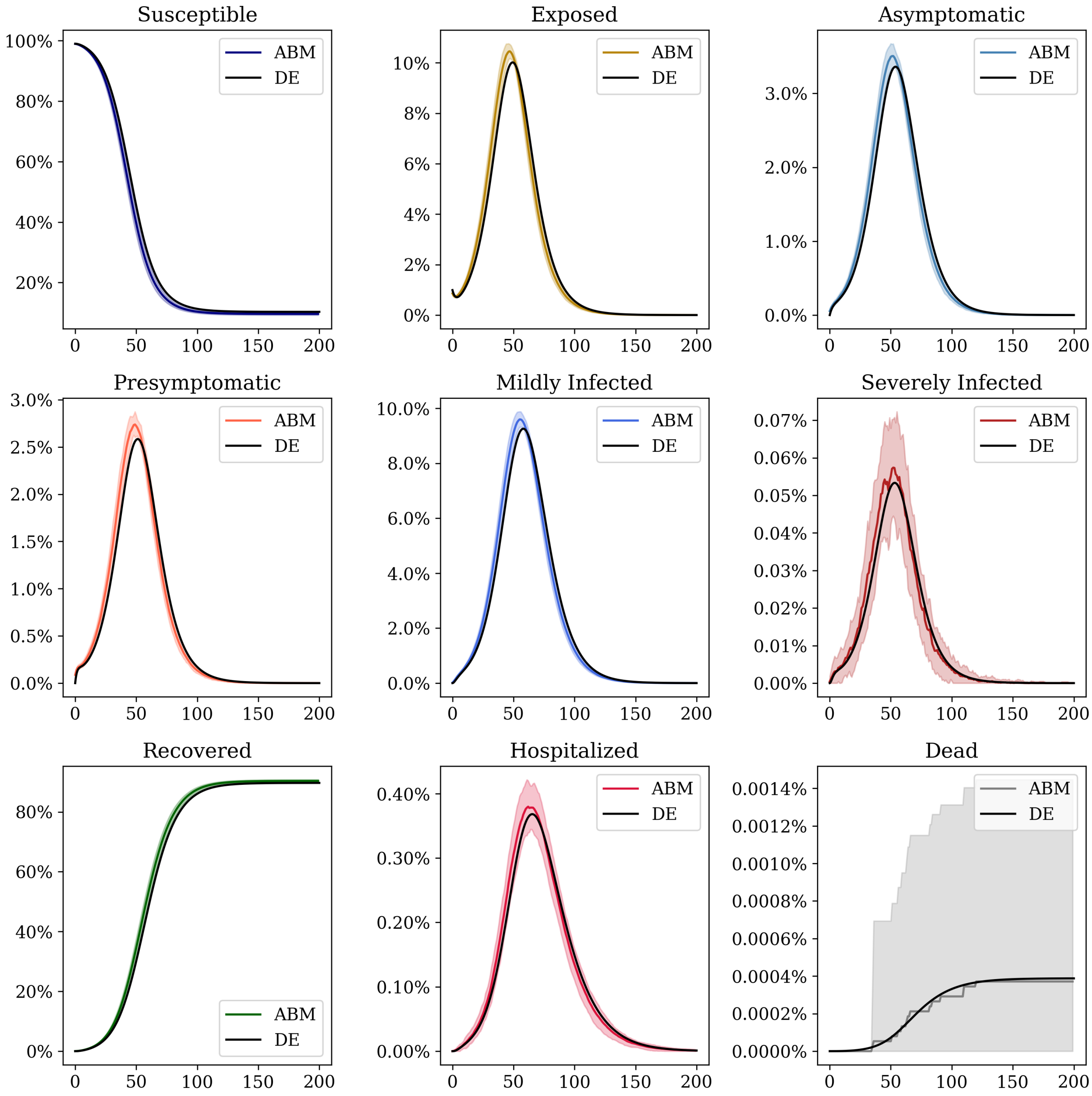

Fig S3.2: Comparing agent-based and differential-equation based models. Epidemic curves for each compartment showing the total infected individuals in the population as a function of time. In each case, a population of 100,000 well-mixed individuals was considered, and 200 simulations were run and averaged over. The shaded regions represent error bars of 1.96σ. The black solid curve shows the same curve for the compartmental model, obtained by solving the differential equations in Eq S1 with the same initial conditions.

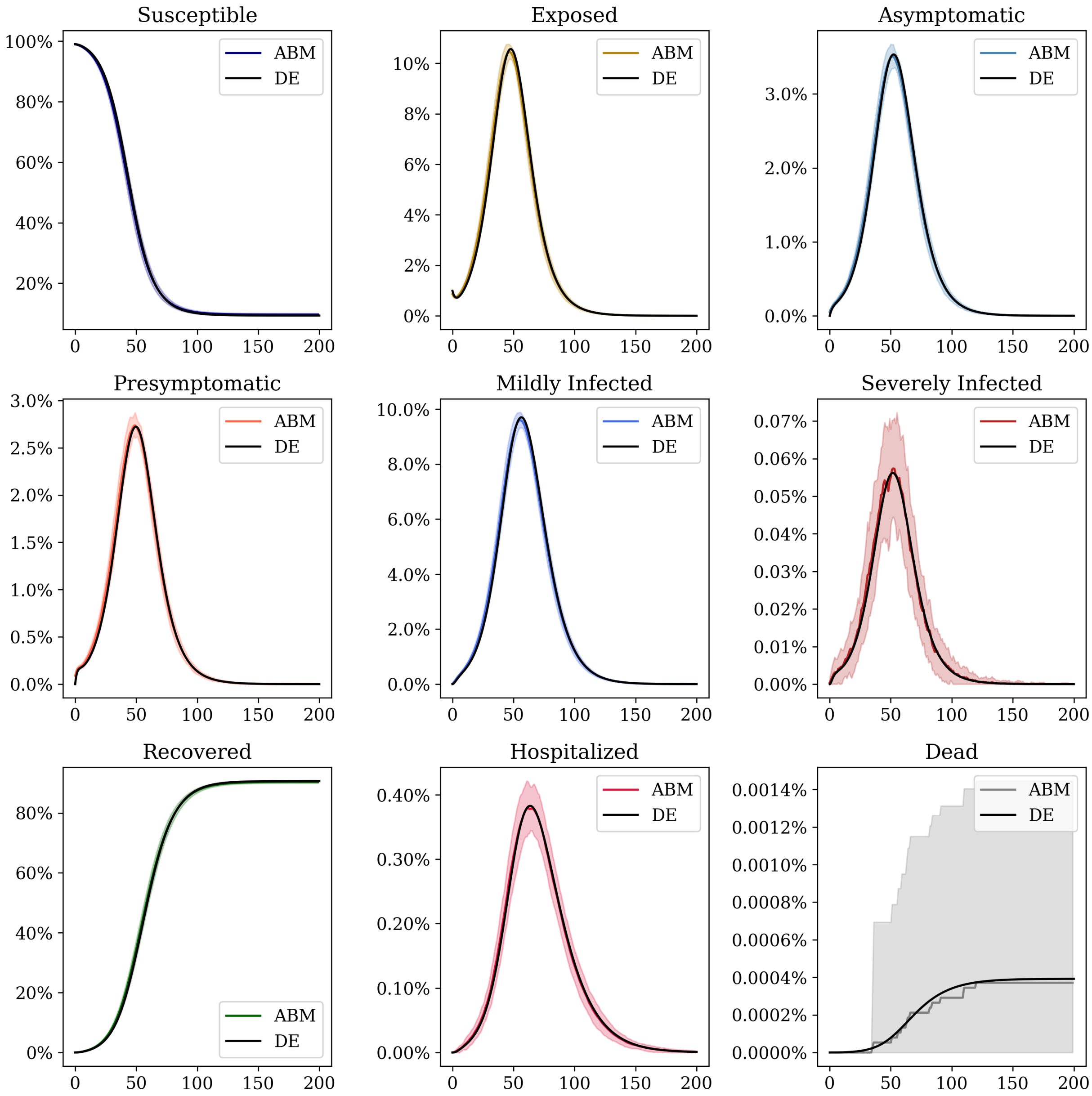

Fig S3.3: Accounting for discrete-time effects. The same graphs as in Fig S3.2, but using a value of $\beta = 0.31$ in the compartmental model. We can see that this small change in β makes all curves from our ABM simulation agree with their compartmental counterparts.
