# Supplementary material for: BharatSim: An agent-based modelling framework for India
Source: PLoS Comput Biol. 2024 Dec 30;20(12):e1012682. doi: 10.1371/journal.pcbi.1012682 (PMC11750085; doi:10.1371/journal.pcbi.1012682)
Supplement: S4 Appendix — We study in detail how sensitive our results are to our model choices. We find that our main results are quite robust to the choice of agents’ travel distances, workplace occupancies, and time spent at home. (PDF) [file pcbi.1012682.s004.pdf]

S4 Appendix: Sensitivity analyses

Here we discuss the sensitivity of the results discussed in the main paper to some of our model choices.

4.1 Sensitivity to home-workplace travel distance

In order to study the role played by the geographical structure of our network, we construct multiple populations for the city of Pune in which individual agents are preferentially assigned workplaces closer to their homes. In Fig S4.1A we show the distribution of the "travel distances" for the agents in our population.

We run simulations with these populations, and for a range of values of the parameter β, which modulates the transmissibility of the disease. In each case we compute the "outbreak size", i.e. the total number of individuals who contracted the disease over the duration of the epidemic. This number is averaged over multiple stochastic runs and the result is plotted in Fig S4.1B. As can be seen from the graphs, the distribution of travel times has a very low effect on the outbreak size. We have also repeated this process for the epidemic curves. We have verified that the difference between them in all the scenarios discussed in the main paper is marginal.

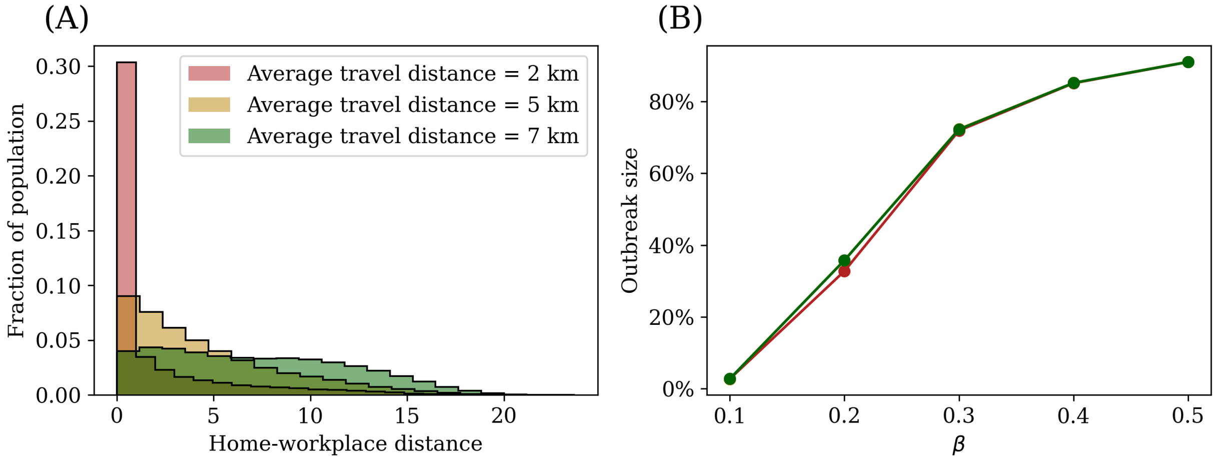

Fig S4.1: Varying individual agents' travel distances. We run our simulations on three different populations in which which we vary the distance that individuals travel between their home and work locations. In (A) we show the distribution of these travel-distances for all agents in each population. Populations in which this distribution is peaked at lower distances are those in which individuals are more likely to interact with other agents who are geographically close to their own homes. In (B) we show the outbreak size as a function of the transmissibility for reach of these populations. As can be seen, as the transmissibility increases, so does the outbreak size. However, we note no significant difference in the results using the different populations. Each data point is the average over 10 simulation runs. Error bars are present at 1.96σ, but are too small to be visible for this population size.

4.2 Sensitivity to workplace occupancy

We further study the role that the distribution of workplace sizes on our results. In order to do this, we consider a population of 100,000 agents with varying average workplace occupancies. We vary both the workplace occupancy and the transmissivity of the disease and compute the outbreak size, as before, and average over multiple stochastic runs. Our results are shown in Fig S4.2. We see that beyond a certain value, the outbreak size is only weakly dependent of the workplace occupancy. Indeed, our results would hold for even workplaces that are as small as 50 agents. We have seen similar results in the literature; compare, for example Fig S4.2B with Fig 4a(iii) in Ref [1].

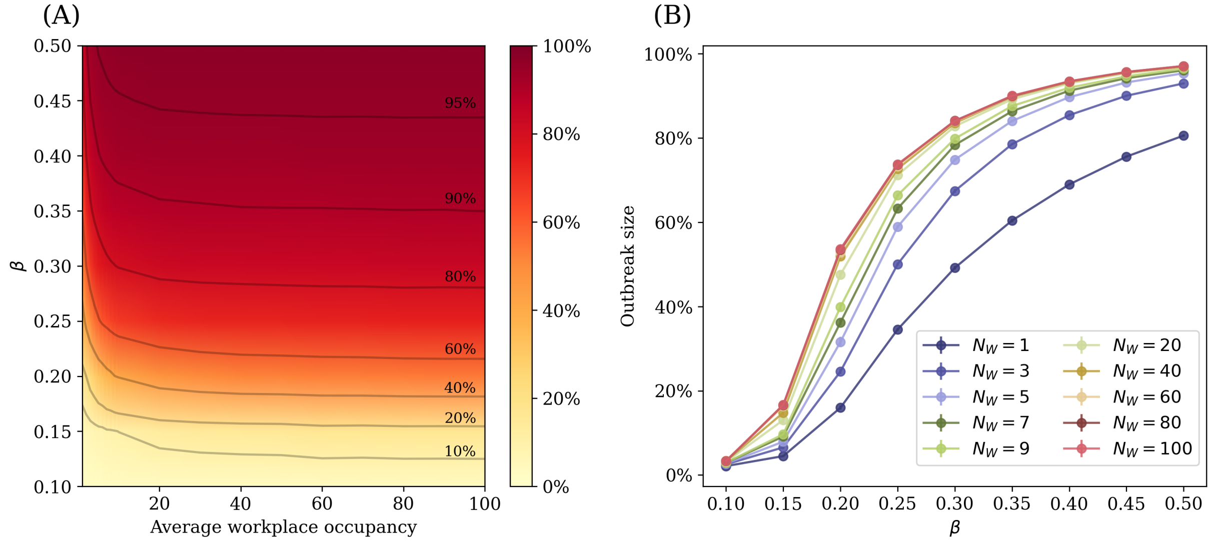

Fig S4.2: Role of workplace occupancy in disease transmission. We study the effect that workplace occupancy has on the transmission of the disease in our models. In (A) we plot a heatmap of the outbreak size as a function of the transmissibility β and the workplace occupancy. Contours are plotted at different outbreak sizes. We see that the outbreak size is much more sensitive to β, and that beyond a workplace occupancy of around 50, the sensitivity to workplace size is very low. In (B) we show a subset of the same results, with each curve representing a single workplace occupancy. Each data point is the average over 200 simulation runs. Error bars are present at 1.96σ, but are too small to be visible for this population size.

4.3 Sensitivity to time spent at home

In order to quantify the sensitivity of our simulations to the time that agents spend at home, we run multiple simulations on a population of 100,000 agents, varying the number of time-steps spent at home. In order to do this, we run simulations in which the time-steps are 6 hours each (four time-steps in a day) and vary the number of steps that individual agents spend at their homes. In Fig S4.3 we show the results for the outbreak size of the simulations. We see that as the amount of time per day spent at home is increased, the outbreak size is reduced, although this is less significant both at low values of tranmissivity β (when the infection dies out relatively quickly) and for high values of β (when the infectious spreads very rapidly though the population), as is intuitively expected.

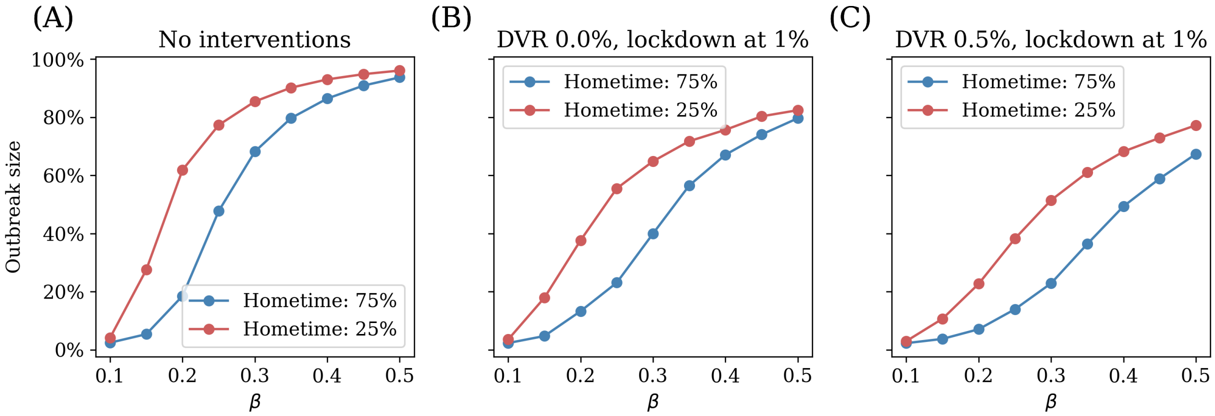

Fig S4.3: Role of time spent at home and work locations on disease spread. We investigate how our results would change based on the repartition of time spent in the low density (home) and high density (workplace) locations. We run multiple simulations with 6-hour time-steps, and vary the number of time-steps spent at home. We show the results here for 1 time-step spent at home (25% of the day, the red curves) and 3 time-steps spent at home (75% of the day, the blue curves). In each case, we compute the outbreak size, and plot it as a function of the transmissibility β. In (A) we show the results for the case where no interventions are applied to the population. In (B) and (C) we show results for when a lockdown is imposed when the number of active cases is 1% of the total population and with no vaccination drive, and with a daily vaccination rate of 0.5%. We see that the introduction of interventions like a lockdown and vaccination drive cause an overall reduction in the outbreak size, as we would expect. Each data point is the average over 50 simulation runs. Error bars are present at 1.96σ, but are too small to be visible for this population size.

References

[1] Hamley JID, Beldi G, Sánchez-Taltavull D. Infectious Disease in the Workplace: Quantifying Uncertainty in Transmission. Bulletin of Mathematical Biology. 2024;86(3):27. doi:10.1007/s11538-023-01249-x.
