# Supplementary material for: BharatSim: An agent-based modelling framework for India
Source: PLoS Comput Biol. 2024 Dec 30;20(12):e1012682. doi: 10.1371/journal.pcbi.1012682 (PMC11750085; doi:10.1371/journal.pcbi.1012682)
Supplement: S5 Appendix — We show how vaccination affects both the relative risks of infection, and of contracting severe disease. These risks are governed by modifying individual agents’ β and α parameters respectively. When an agent receives a dose of the vaccine, their protection increases linearly over a 14 day window, until it plateaus to the maximum protection offered by that dose. The reduction in the relative risk of infection (i.e. in β) is assumed to be the same for each age-group, while the reduction in the risk of contracting severe disease is age-stratified. (PDF) [file pcbi.1012682.s005.pdf]

S5 Appendix: The effect of vaccination

We consider vaccination as having three distinct effects: a reduction in the relative risk of infection, a reduction in the probability of severe infection, and a lower probability of transmitting the disease.

Vaccinated individuals are assumed to be 40% less likely to transmit the disease from the day they receive their first dose. The other relative effects evolve as a function of time. In both cases, we assume that the maximum protection from each dose is attained within 14 days of receiving the dose. In the interim period, we assume each parameter varies linearly. Fig S5.1A shows the reduction in the relative risk of infection, and Fig S5.1B shows the increase in the probability that a vaccinated individual will not exhibit symptoms.

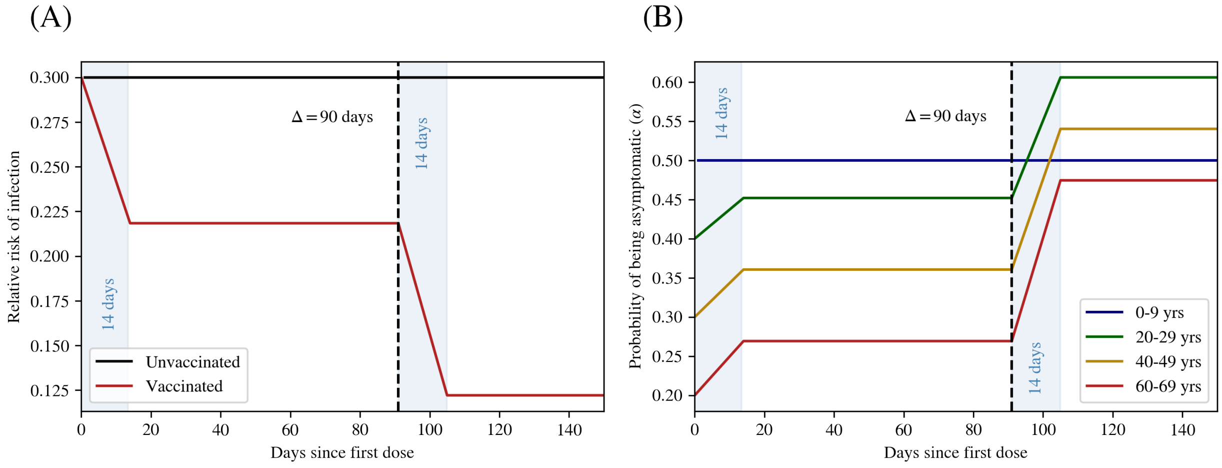

Fig S5.1: Variation of relative risks of infection and severe disease as a function of time. After each vaccine dose, these parameters reduce over a period of 14 days until the maximum protection from that dose is obtained. The same process is repeated after the second dose is obtained. (A) Reduction in the relative risk of infection reduces over a period of 14 days. (B) Increase in the probability of being asymptomatic.
