# Supplementary material for: BharatSim: An agent-based modelling framework for India
Source: PLoS Comput Biol. 2024 Dec 30;20(12):e1012682. doi: 10.1371/journal.pcbi.1012682 (PMC11750085; doi:10.1371/journal.pcbi.1012682)
Supplement: S6 Appendix — The vaccine doses are distributed between first- and second-shot vaccinations in an 80:20 ratio, in such a way as to minimize dose-wastage, as discussed in the main text. We discuss the algorithm used to enforce this, and illustrate through examples how the doses are distributed in a population of 10,000 agents for different daily vaccination rates. (PDF) [file pcbi.1012682.s006.pdf]

S6 Appendix: Vaccine dose prioritization and avoiding wastage

The number of available vaccine doses that can distributed each day is decided by the daily vaccination rate. The allocation of vaccine doses is done by assuming some prior dose prioritization: some fraction τ of the total doses (chosen in our simulations to be 80%) are designated as "first" doses, and distributed amongst those individuals who are eligible to receive their first vaccine dose. The remaining fraction $(1-\tau)$ of doses are used to vaccinate individuals who are eligible for the second vaccine dose. However, this fraction is adjusted dynamically such that dose wastage is minimized.

At every time-step, a fixed number of individuals (set by the total number of available vaccines) is chosen from those who are eligible for the first shot of the vaccine. Individuals are eligible for vaccination provided (i) their age-band is one of those being vaccinated during the current phase of the vaccination drive, (ii) they have not yet received this specific dose, and (iii) the individual is not exhibiting symptoms. The same process is repeated for the second dose. For the second dose of the vaccine, in addition to the earlier eligibility criteria, at least Δ days must have elapsed since the individuals received their first dose. In our simulations we choose $\Delta = 90$.

Once these individuals are chosen, fractions τ and $(1-\tau)$ of the vaccines are administered to those eligible to receive the first and second doses respectively. If there are any leftover vaccines (for example, there are more first doses available that eligible individuals), we then evaluate the excess doses for both first and second shots, swap them, and re-administer. In other words, we use any excess second doses to vaccinate those eligible for their first dose and vice versa. While the resulting ratio will not necessarily be τ, this ensures that all available doses are used in a single day, unless there are no eligible people in the population for any vaccine dose.

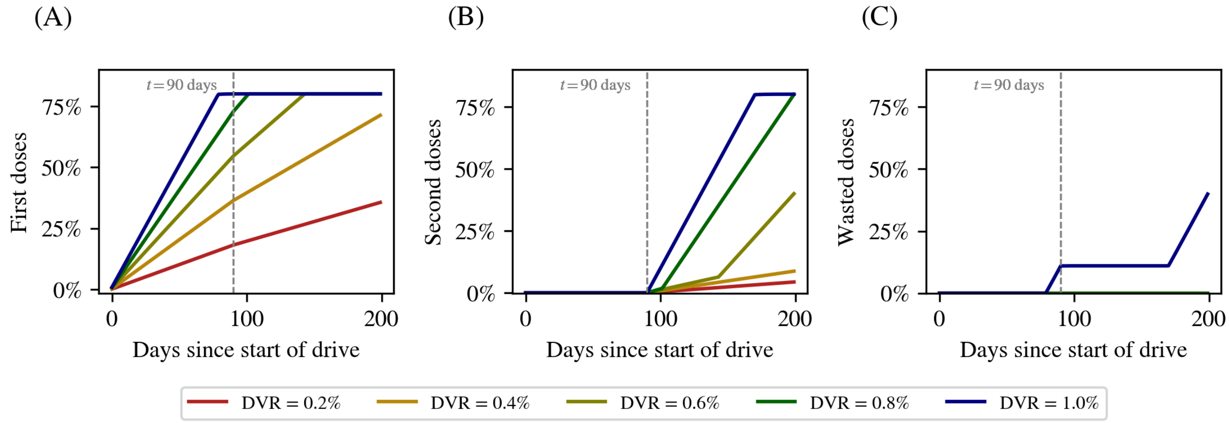

Fig S6.1: First, second, and wasted doses as a function of time. The fraction of the total population that are vaccinated with (A) first and (B) second doses for daily vaccination rates from 0.2% to 1%. In all cases, the vaccine drive starts on day 0. The dose-prioritization ratio is 80:20, with 20% of the doses being designated as second-doses. For the first 90 days, all doses administered are first-doses, since no one is eligible for a second dose, after which second doses start getting administered. The numbers plateau at a value less than 100% since not everyone in the population is eligible for a vaccine. In (C) we show the number of wasted doses, again as a fraction of the total population. In the case of DVR = 1%, the vaccination rate is so high that there are occasionally more doses available than eligible agents, leading to dose wastage.
