# Supplementary material for: BharatSim: An agent-based modelling framework for India
Source: PLoS Comput Biol. 2024 Dec 30;20(12):e1012682. doi: 10.1371/journal.pcbi.1012682 (PMC11750085; doi:10.1371/journal.pcbi.1012682)
Supplement: S7 Appendix — We describe two of the populations used to obtain our simulation results. The first is a population for the entire city of Pune with 3.13 million individuals. The second is a section of the synthetic population for the city of Pune, which is chosen with 20,316 individuals with 6500 homes, 120 workplaces, and 1 school. (PDF) [file pcbi.1012682.s007.pdf]

S7 Appendix: Descriptions of the synthetic populations used in our simulations

We describe the synthetic population used in our simulations for the city of Pune, and for the study of school reopenings.

7.1 Metrics for the synthetic population of Pune

In Fig S7.1 we show the geographical distribution of homes, workplaces, and schools.

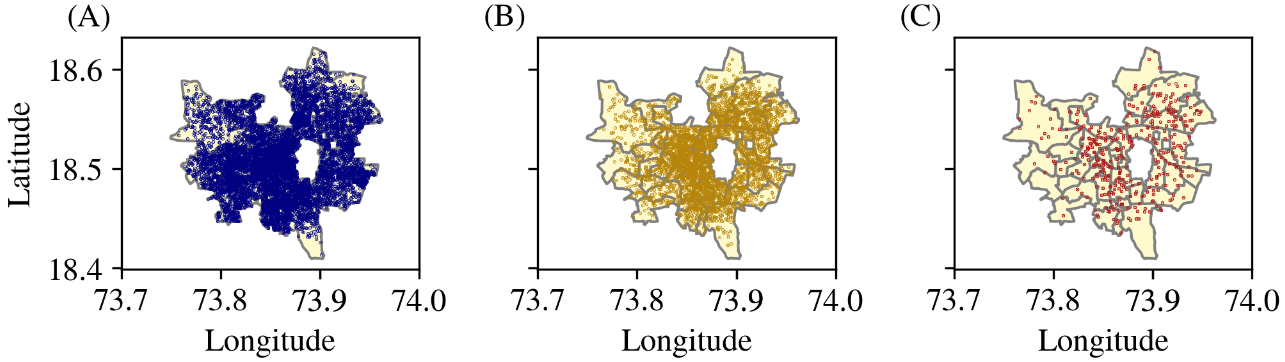

Fig S7.1: Distribution of geo-locations for the city of Pune. Geographical distribution of (A) households, (B) workplaces, and (C) schools for the combined synthetic population of the districts of Pune. The underlying map of Pune is provided by the Spatial Data of Municipalities (Maps) Project by Data{Meet} [1].

In Fig S7.2, we compare the distribution of ages in the survey and the synthetic population of Pune. We work with a randomly chosen subset of the synthetic population comprising 10,000 individuals to compare with the survey data, although our full synthetic population has over 3 million individuals.

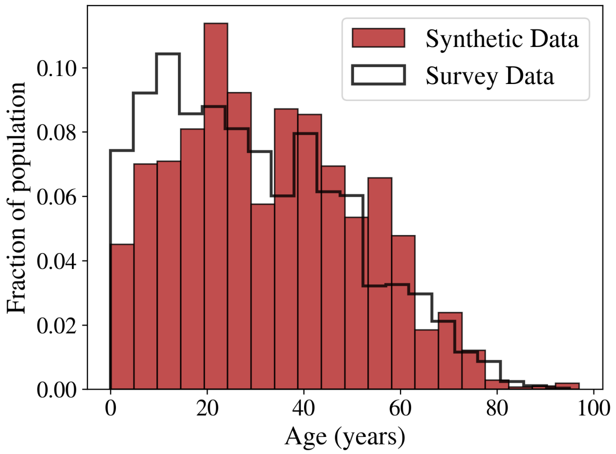

Fig S7.2: Histogram of age distribution. The distributions of age in the synthetic population for the population of Pune for both the synthetic population used in the modelling results of the main paper, and data from the IHDS-II survey.

We also compute statistical metrics, as described in Appendix S2, to compare our population with the IHDS-II dataset for Maharashtra. The results are shown in Tables S7.1 and S7.2.

Features	Boundary Coverage	RangeCoverage	StatisticSimilarity	KSComplement
Height	1.0000	0.9000	0.9800	0.9000
Weight	1.0000	0.6300	0.9600	0.7400
Age	1.0000	1.0000	0.9700	0.8900

Feature	TVComplement
SexLabel	0.9200
M_Cough	0.9980
M_Cancer	0.9990
M_Diarrhea	0.9990
M_Fever	0.9990
M_Cataract	0.9980
M_TB	1.0000
M_HeartDisease	0.9990
M_Diabetes	0.9990
M_HighBP	0.9990
M_Leprosy	0.9990
M_Asthma	0.9990
M_Paralysis	0.9990
M_Epilepsy	0.9980
M_Polio	1.0000

Table S7.1: Metrics for comparing numerical (age, height, and weight) and categorical (comorbidity) columns between the Pune synthetic population used in our simulations and the IHDS-II survey data. In every test a result of 1.0 signifies strong correlation and 0.0 signifies no correlation between the survey and synthetic data.

7.2 Description of the school population

A section of the synthetic population for the city of Pune is chosen with 20,316 individuals with 6500 homes, 120 workplaces, and 1 school. These individuals represent the catchment area for the school. In Fig S7.3 we show the geographical distribution of the school population, and in Fig S7.4 we show some of its statistics.

References

[1] Data{Meet} Community. Spatial data of Municipalities; 2024. Available from: http://projects.datameet.org/Municipal_Spatial_Data/.

Features	CorrelationSimilarity
Age, Height	0.9900
Age, Weight	0.9800
Height, Weight	0.9500

Features	ContingencySimilarity
M_Cough, M_HeartDisease	0.9980
M_Diabetes, M_HeartDisease	0.9990
M_Cough, M_Fever	0.9980
M_Cough, M_Asthma	0.9980

Table S7.2: Metrics for comparing the joint distributions of numerical (age, height, and weight) and categorical (comorbidity) columns between the Pune synthetic population used in our simulations and survey data from IHDS-II. In every test a result of 1.0 signifies strong correlation, and 0.0 signifies no correlation, between the survey and synthetic data.

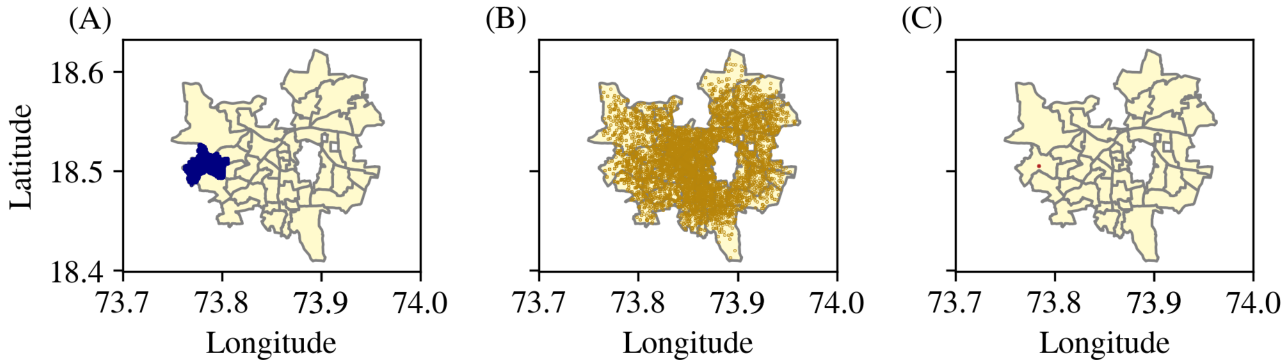

Fig S7.3: School synthetic population geographical distribution. The distributions of (A) households, (B) workplaces, and (C) (one single) school for this section of the population. The underlying map of Pune is provided by the Spatial Data of Municipalities (Maps) Project by Data{Meet} [1].

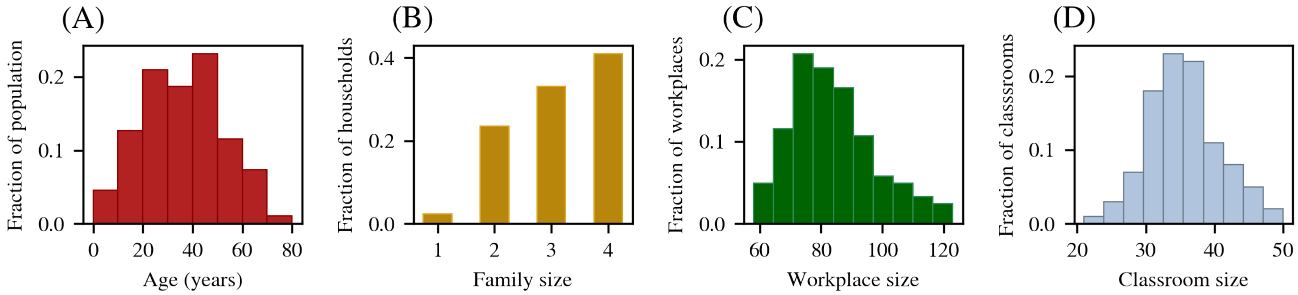

Fig S7.4: School synthetic population statistics. The distributions of (A) age, (B) family size, (C) workplace size, and (D) classroom size for this section of the population.
