# Supplementary material for: BharatSim: An agent-based modelling framework for India
Source: PLoS Comput Biol. 2024 Dec 30;20(12):e1012682. doi: 10.1371/journal.pcbi.1012682 (PMC11750085; doi:10.1371/journal.pcbi.1012682)
Supplement: S8 Appendix — We compare our model projections with real data for confirmed cases from the initial part of the first wave of COVID-19 in the city of Pune. These projections, and an understanding of how they are modulated by the interventions made, can then be directly used for public health planning in an epidemic situation. (PDF) [file pcbi.1012682.s008.pdf]

S8 Appendix: Using BharatSim for epidemic forecasts: a case study for Pune

In this section, we describe how BharatSim can be used for epidemic forecasts. We use real data for confirmed cases from the initial part of the first wave of COVID-19 in the Indian city of Pune. In the period we consider, the city was twice placed under a rigorous lockdown, with an intervening period of several weeks when the lockdown was lifted.

Here we demonstrate the versatility of BharatSim by comparing model projections based on initial independent estimates of the basic reproductive ratio R_0, to real data. The collective network properties of agent interactions are modified by public health interventions such as lockdowns. The projections, and an understanding of how they are modulated by the interventions made, can then be directly used in public health planning in an epidemic situation.

We first calibrate the value of β used in our simulations to match the initial R_0. We obtain R_0 from a statistical analysis of daily case data [1]. These estimates can be further refined as time proceeds.

8.1 Computing the reproductive ratio from our simulations

The basic reproductive ratio is defined as the average number of people infected by a single contagious person in the background of a population of susceptible people. Its value, in an averaged sense, depends on the mean number of contacts, the probability that an infection will result from any given contact over the period of the interaction, and the time over which an individual remains infected. In our agent-based simulations, network structure plays an important role in determining R_0.

To compute this quantity from our simulation results, we keep a record of which agent was responsible for each infection. Whenever an individual is infected in a specific location, an agent is drawn at random from the potential "infectors" at that location so that infection spread can be described.

The simulations are run for a range of values of β, for a period of 21 days. This period is chosen so that a sufficient number of infectious individuals recover. At the end of this period, the average number of people infected by a person who has recovered from the disease, is computed. These results are shown in Fig S8.1. We conclude from this figure that if we require a value of R_0 to be around 1.2–1.3, as estimated in the statistical analysis of case data for this period (see Section 6.4 in the Supplementary Information of Ref [2]), a value of $\beta = 0.4$ should be used in our simulation.

8.2 Predicting the spread of COVID-19 in the first wave

We begin our simulations with our population in a lockdown state. The initial lockdown in Pune was imposed from the 25th of March until the 31st of May, 2020. During this period, the agents in our simulation were allowed to have only limited mobility – only those marked as "essential workers" were allowed to move out of their homes. We began the simulation by seeding 1500 exposed agents in the population, distributed randomly around the city. After the 31st of May, the lockdown was lifted, and agents allowed to move between their homes and workplaces. This led to an increase in the spread of the disease. On the 14th of July, a second ten-day lockdown was imposed in Pune.

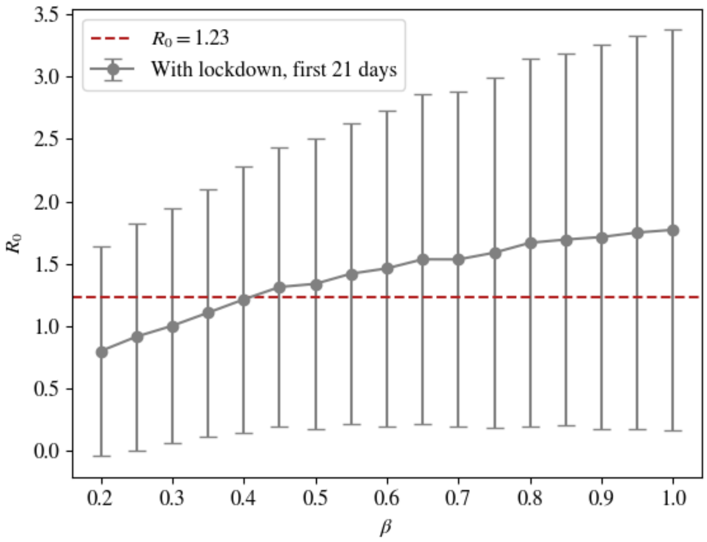

Fig S8.1: The basic reproductive ratio obtained from our simulation as a function of the transmission factor β. We see that in order to obtain an R_0 in the range 1.2 to 1.3, a value of $\beta = 0.4$ must be used.

During the entire simulation, schools remained closed, as was the situation across the first wave. Our simulations thus capture the actual interventions that were applied, and can be used to model the response in the numbers of infected to those interventions.

8.3 Results

In Fig S8.2 we show our results by comparing the number of daily recorded cases with the number of active symptomatic cases in the population, corrected for under-counting. (Due to major limitations in testing during much of the first wave of COVID-19 in India, as well as social stigma associated with reporting infections, it is believed that case under-counting was substantial in the first several months of the spread of COVID-19 in India.) We account for case under-counting by dividing the total number of active symptomatic cases estimated in the simulation by a constant factor of 150; this is consistent with national estimates for India in the same period.

Fig S8.2 shows that, with our initial conditions, we are able to predict the spread of the disease quite accurately until the end of the second lockdown, given only (a) an estimate for initial infections, (b) a single number representing the over-counting factor and (c) a value of R_0 extracted from a time-series analysis of the first few weeks of reported cases. It is difficult to project beyond this, since the mix of variants changed decisively across the month of August 2020 [3,4], and reinfections arising from waning immunity from a prior infection (which we excluded in this analysis) became important. Nevertheless, our methods capture the rise in cases after the lockdown was lifted, the peak in cases during the second lockdown and the subsequent decrease in case numbers, attesting to the value of well-bench-marked agent-based models in providing real-time information regarding the trajectory of infections during an ongoing epidemic.

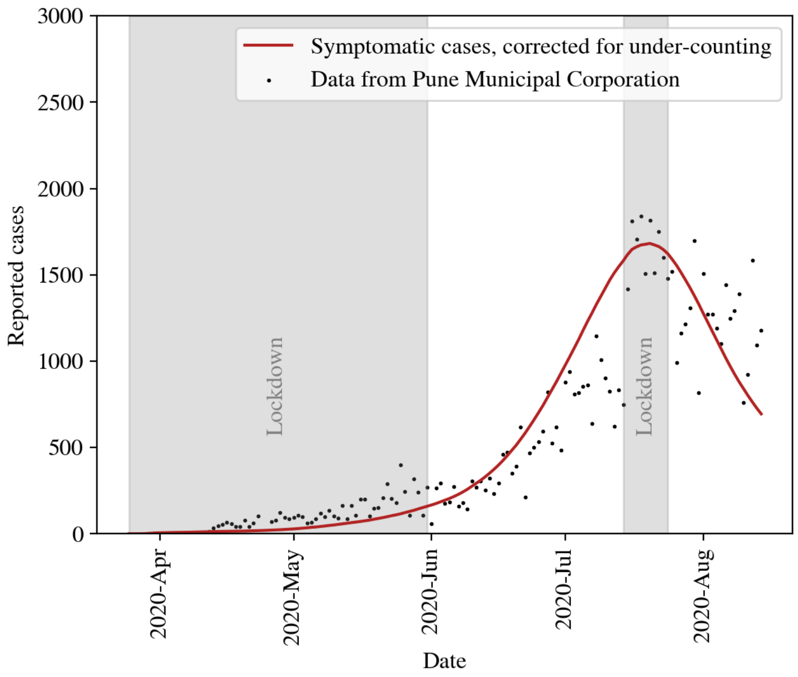

Fig S8.2: Our simulation results compared with data from the Pune Municipal Corporation. Using the value of $\beta = 0.4$ and an initial number of 1500 exposed agents, we are able to predict the spread of the disease, assuming a constant under-counting factor of 150. Our curves are obtained by looking only at the number of individuals in the symptomatic compartments of our model. If all infected individuals had been used, a similar result could be obtained, albeit with a higher under-counting factor.

References

[1] CESSI. CESSI COVID-19 Dashboard; 2023. Available from: http://www.cessi.in/coronavirus/index.html.

[2] Hazra DK, Pujari BS, Shekatkar SM, Mozaffer F, Sinha S, Guttal V, et al. The INDSCI-SIM model for COVID-19 in India. medRxiv; 2021. Available from: https://www.medrxiv.org/content/10.1101/2021.06.02.21258203v1.

[3] Wahengbam R, Bharali P, Manna P, Phukan T, Singh MG, Gogoi G, et al. Seroepidemiological and genomic investigation of SARS-CoV-2 spread in North East region of India. Indian Journal of Medical Microbiology. 2023;43:58–65. doi:10.1016/j.ijmmb.2022.10.011.

[4] Pandit B, Bhattacharjee S, Bhattacharjee B. Association of clade-G SARS-CoV-2 viruses and age with increased mortality rates across 57 countries and India. Infection, Genetics and Evolution. 2021;90:104734. doi:10.1016/j.meegid.2021.104734.
