# Supplementary material for: BharatSim: An agent-based modelling framework for India
Source: PLoS Comput Biol. 2024 Dec 30;20(12):e1012682. doi: 10.1371/journal.pcbi.1012682 (PMC11750085; doi:10.1371/journal.pcbi.1012682)
Supplement: S9 Appendix — We use BharatSim to model the spread of mpox, a viral infection caused by a zoonotic virus in the genus Orthopoxvirus. A global outbreak of mpox associated with sexual contact has been ongoing since May 2022, with the vast majority of cases being diagnosed among men who have sex with men (MSMs). Here, we describe a possible use of BharatSim to study the spread of mpox in a population of 10,000 individuals that is meant to represent a group of MSMs and their contact networks. (PDF) [file pcbi.1012682.s009.pdf]

S9 Appendix: Studying the spread of mpox

Mpox is a viral infection caused by the mpox virus, a zoonotic virus in the genus *Orthopoxvirus*. Concerned by the sudden global spread of mpox beyond those African countries where it had previously been reported, the WHO declared it a Public Health Emergency of International Concern (PHEIC) in July, 2022. In the period between 01 January, 2022, and August 31, 2024, 123 countries have reported a total of 1,06,310 laboratory confirmed mpox cases and 234 deaths [1]. In India, 30 mpox cases have been reported since the WHO's 2022 PHEIC declaration [2].

Prior to the current outbreak, mpox infections were generally thought to arise mainly from contact with animal reservoirs. However, human-to-human transmission through direct routes (skin-to-skin contact, bodily fluids, and respiratory droplets) have also been recorded. Transmission could plausibly occur through sexually associated exposure to skin lesions, droplets, and fomites [3]. The 2022 outbreak of mpox was primarily associated with close intimate contact (including sexual activity) and most cases were diagnosed among men who have sex with men (MSMs), with 98% of the patients in a report of 528 cases from 16 countries being MSMs [4, 5].

In this section, we use BharatSim to model the spread of mpox in a community of MSMs and their associated household and workplace contacts.

9.1 Creating a synthetic population

Since granular data for sexual contacts for MSMs is hard to obtain (since it might place individuals belonging to a stigmatised population at risk), our model bases itself on reconstructing sexual networks from aggregate data made available in [6] and [7].

As in the case of our simulations for multiple strains in the main paper (Section 3.3), we consider a population of 10,000 individuals, which we take to represent both MSMs and the network of their contacts. In principle, this could have been done with the BharatSim synthetic population directly, but the simpler procedure described here suffices to extract features of mpox spread in model populations with contact patterns similar to those characterised in the Indian context, without the overhead of the many additional (and for these purposes, irrelevant) variables that enter the description of the BharatSim population.

Of these 10,000 individuals, 1% are assumed to be MSM, while the others are their household and workplace contacts. The households and workplaces are distributed so that their mean occupancies are 4 and 50 respectively. This is done by first creating 2500 homes (corresponding to an average household size of 4 individuals) and assigning each individual to one of them with a uniform probability. The same process is repeated with workplaces, ensuring the correct average number of agents per location, distributed as a Poisson distribution.

9.2 Assigning a contact network for MSMs

A quantity of some importance is the network of MSMs in the population. Studies have shown [3] that the sexual-networks of MSMs are often heavy-tailed, and are reasonably well modelled by distributions like the Weibull distribution. We use the range of number of partners shown in Ref [6], modelling this through a Weibull distribution, where the lowest and highest values in this range correspond to the 10% and 90% percentile respectively. The MSM network is created with this degree distribution in the following way:

- First, a trial number of contacts is chosen from the list of MSM agents. These numbers will be the starting point for our final distribution of contacts.
- Next, a list of all agents is made, with each agent being replicated as many times as they have contacts. This list is then shuffled to avoid the same agent being placed next to itself.
- Last, successive pairs of agents in the newly shuffled list are paired together, as far as possible, while avoiding mapping an agent as their own contact.

This method provides networks whose statistical properties are consistent with those available in the literature for MSM interactions.

The resulting distribution of contacts for MSMs and their graph is shown in Fig S9.1. This should be compared to similar figures in Ref [6].

9.3 The disease progression

Mpox is modelled using compartments from an $SEIR$ model, with Susceptible, Exposed, Infected, and Removed individuals. The incubation period for the disease is assumed to be 7 days, and the infectious period to be 21 days (following, for example, Refs [3] and [8]). Sojourn times in each of these compartments are assumed to follow an exponential distribution.

Agents can transmit the disease amongst each other in one of two channels: either sexually (for MSMs) or non-sexually (for everyone). We assume that the greatest transmission of the disease between agents via non-sexual contacts occurs at home. The parameter β controls the force of infection at the home. This parameter is varied, to see how the spread of the disease varies with the disease transmission strength. In addition, the model also allows for a weak probability of transmission in the workplace as well, assuming a reduced level of physical contact leading to infection.

In addition to individuals moving between homes and workplaces in 12 hour schedules, MSM agents in our simulations can also meet other agents in their sexual contact network through a sexual "encounter". This is modelled in the BharatSim framework as a "`behaviour`" (see Section 2.2 of the main text). The probability distribution of the interval between which an individual MSM meets a contact in his network is assumed to be an exponential distribution with a mean of 7 days.

During such a sexual encounter, if either of the two MSMs is infected, they can infect the other, with some probability μ. In the absence of empirical data on this parameter, we vary μ from 0–100% in our simulations, as done in Ref [3]. Other modelling studies (see Ref [8], for example) assume a single value of 20%. We note that μ can also be used to model the usage of prophylactic measures like condoms, which have been found to reduce the risk of mpox spread [9].

9.4 Results

We simulate the spread of the disease by assuming an initial infection seed of a single MSM in the population. In Fig S9.2 we show the total infection curves for different values of the transmission parameter β and the probability of infection per sexual encounter μ. The different curves in each of the panels represent different values of μ. The figures show the average results over 100 simulations, but it is worth noting that there is substantial variance, with a good portion of runs dying out without percolating to the entire network.

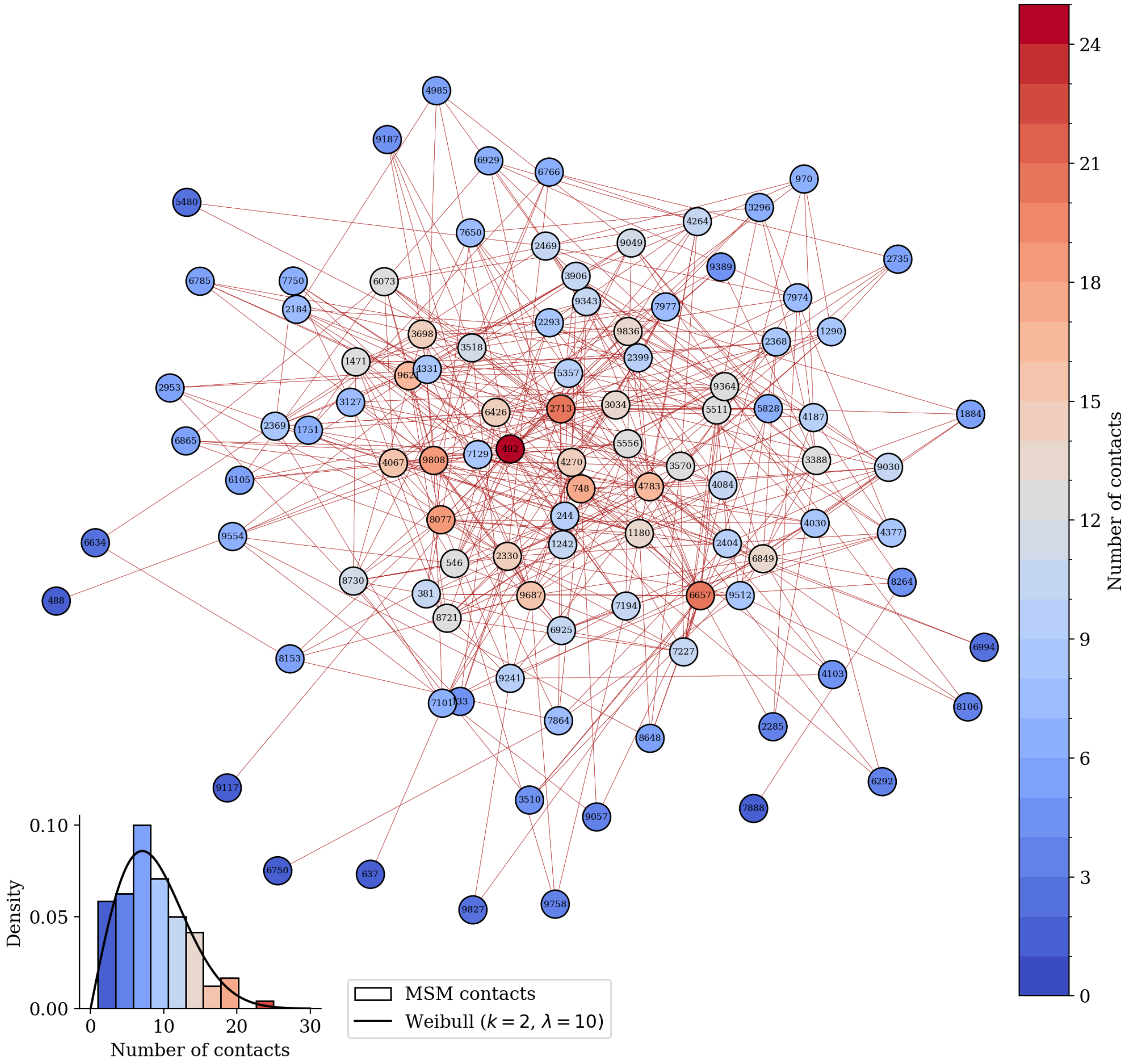

Fig S9.1: The contacts of the MSMs in the population as assumed to be distributed as a Weibull distribution. The statistics of the distribution were chosen such that they fall within the range of the results shown in [6]. In the inset we show the actual distribution of contacts, compared with a fitted Weibull distribution with parameters $k = 2, \lambda = 10$.

We then explore the possibility of getting similar results by starting with an initial infection seed involving non-MSM agents. We find that in order to get an outbreak size that is comparable, we require a much higher initial infection seed; this has also been seen in related work [3]. In Fig S9.3, we run a similar set of simulations which we seed with 50 non-MSM agents. Interestingly, the initial spread of the disease is dominated by the non-MSM population. During this spread there is no dependence on μ. However, once the disease enters the MSM population, a strong dependence on μ is seen, and a second epidemic peak can be observed, whose dynamics is driven by the MSM population.

In addition, if we now include a very small probability of infection at the workplace (1% the rate of infection in the household), these effects are further amplified, as shown in Fig S9.4.

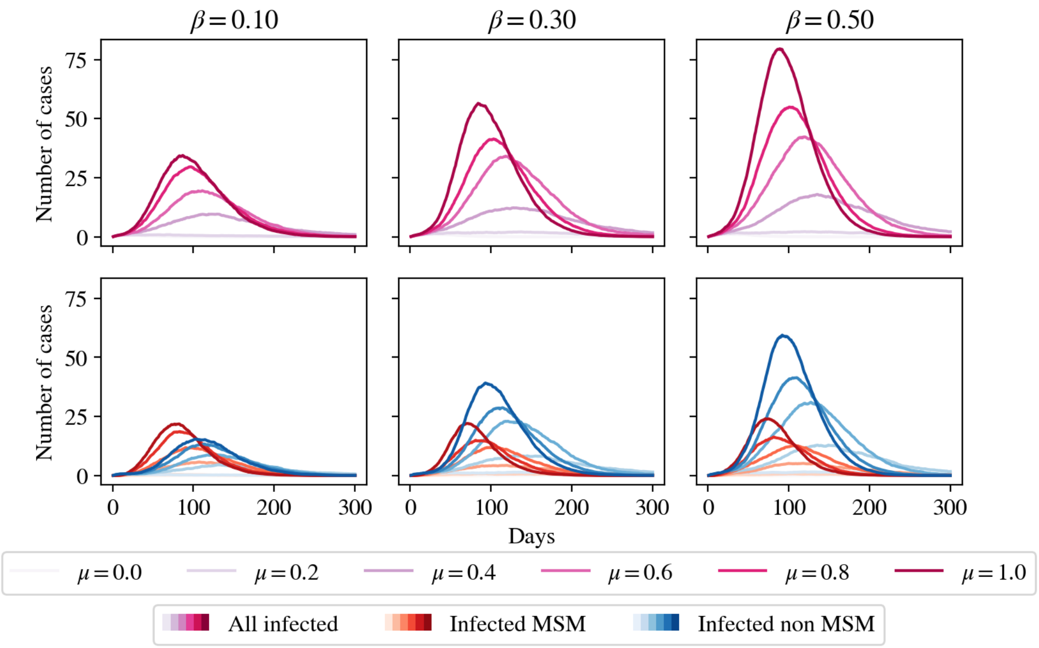

Fig S9.2: Infection curves for different values of household infectivity β and probability of sexual transmission μ, when the infection is seeded with 1 MSM agent. The top row of panels shows the the total number of active infections in the population in each scenario, while the bottom row shows the division of cases between the MSM and non-MSM population. The red curves show the spread in the MSM population, while the blue curves show the spread in the general population. Darker colours indicate higher values of μ. We see that the peak in the general population is shifted further to the right as compared to the MSM peak. Additionally, we find that the MSM peak is more or less independent of the parameter β, when compared to the general population peak, however both of these peaks depend strongly on the parameter μ. The curves are averages over 100 runs.

9.5 Discussion

Our results illustrate how the movement of the infection between MSM and non-MSM networks can lead to complex structure in the progress of infection, involving at least two time-scales. Notably, the spread initiated in the non-MSM population can produce an initial peak that can then give rise to a second peak once it enters the MSM network. We also illustrate how a long tail of infection can be produced as a consequence of even weak spread involving transmission in the workplace. This has clear implications for control.

The results here may be useful to public health policy as it pertains to mpox control in the Indian context, especially since the effectiveness of prophylactic measures such as vaccinations, applied to different sections of the population, can be assessed using BharatSim.

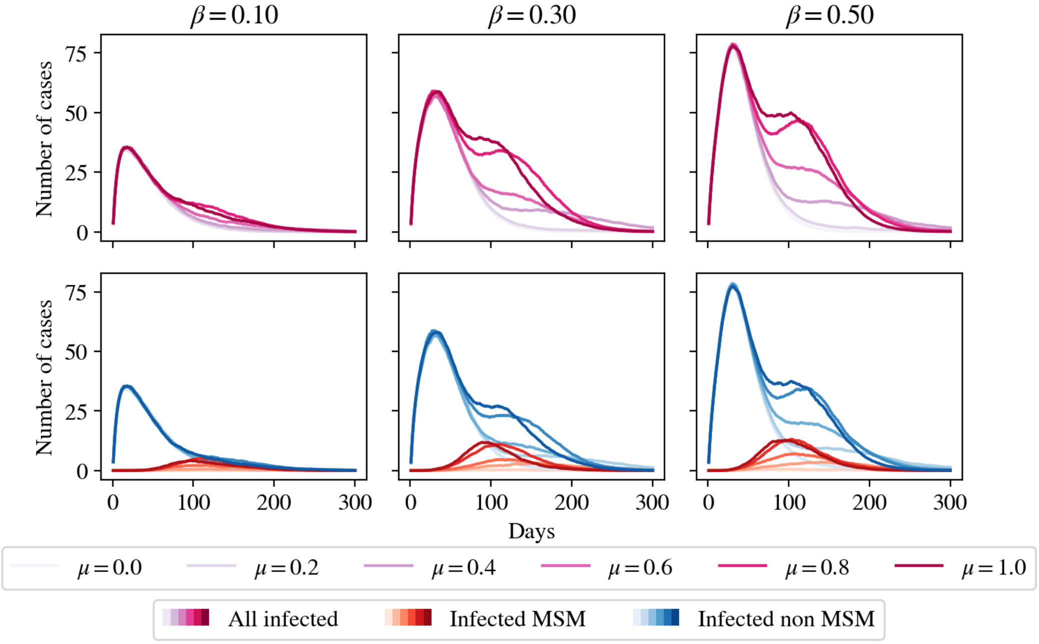

Fig S9.3: The same results as in Fig S9.2, but with an initial infection seed of 50 non-MSM agents. We find that this higher initial infection seed is needed to get comparable outbreak sizes. While the initial spread of the disease is amongst the non-MSM population and therefore independent of μ, we see that a secondary peak is observed once the disease enters the MSM network, post which a strong dependence on μ is found. Again, the curves are averages over 100 runs.

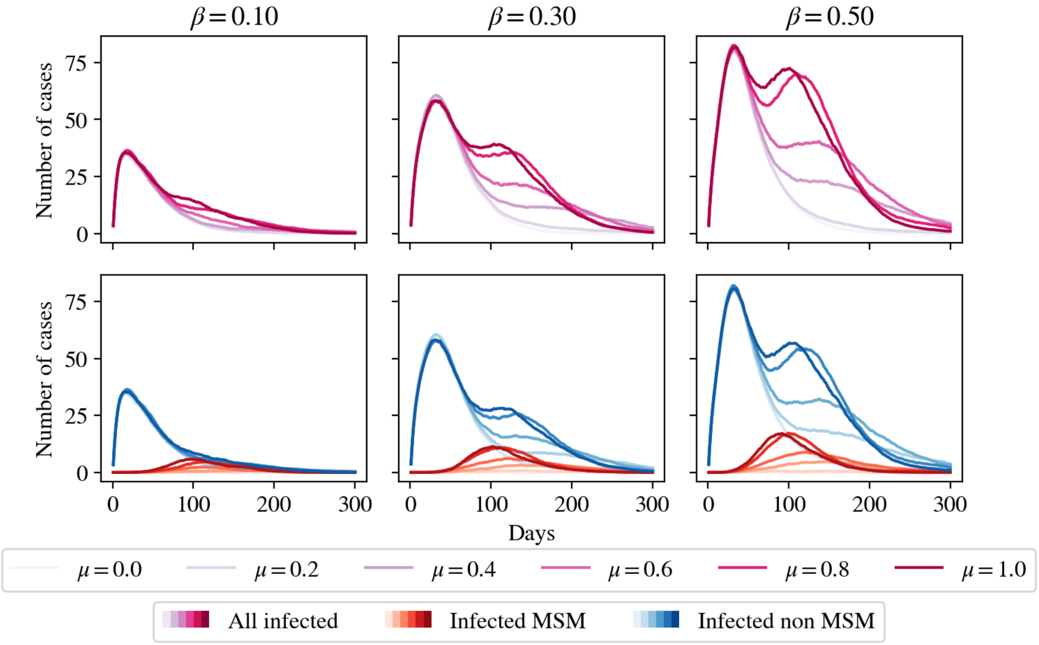

Fig S9.4: Continuing the assume an initial infection seed of 50 non-MSM agents, we find that if we include a small probability of being infected in the workplace (1% the probability of household infection), the results in Fig S9.3 are further amplified. The curves are averages over 100 runs.

References

[1] World Health Organization. 2022-24 Mpox (Monkeypox) Outbreak: Global Trends; 2024. Available from: https://worldhealthorg.shinyapps.io/mpx_global/.

[2] Kumar S, Kataria R, Sharma A. India Against Mpox; 2024. Available from: http://pib.gov.in/PressNoteDetails.aspx?NoteId=152119.

[3] Endo A, Murayama H, Abbott S, Ratnayake R, Pearson CAB, Edmunds WJ, et al. Heavy-tailed sexual contact networks and monkeypox epidemiology in the global outbreak, 2022. Science. 2022;378(6615):90–94. doi:10.1126/science.add4507.

[4] Mitjà O, Ogoina D, Titanji BK, Galvan C, Muyembe JJ, Marks M, et al. Monkeypox. The Lancet. 2023;401(10370):60–74. doi:10.1016/S0140-6736(22)02075-X.

[5] Thornhill JP, Barkati S, Walmsley S, Rockstroh J, Antinori A, Harrison LB, et al. Monkeypox Virus Infection in Humans across 16 Countries — April–June 2022. New England Journal of Medicine. 2022;387(8):679–691. doi:10.1056/NEJMoa2207323.

[6] Lorway R, Shaw SY, Hwang SDH, Reza-Paul S, Pasha A, Wylie JL, et al. From individuals to complex systems: exploring the sexual networks of men who have sex with men in three cities of Karnataka, India. Sexually Transmitted Infections. 2010;86 Suppl 3:iii70–78. doi:10.1136/sti.2010.044909.

[7] Satyanarayan S, Kapur A, Azhar S, Yeldandi V, Schneider JA. Women Connected to at Risk Indian Men Who Have Sex with Men: An Unexplored Network. AIDS and Behavior. 2015;19(6):1031–1036. doi:10.1007/s10461-014-0946-1.

[8] Van Dijck C, Hens N, Kenyon C, Tsoumanis A. The Roles of Unrecognized Mpox Cases, Contact Isolation and Vaccination in Determining Epidemic Size in Belgium: A Modeling Study. Clinical Infectious Diseases. 2023;76(3):e1421–e1423. doi:10.1093/cid/ciac723.

[9] Chard AN. Risk of Clade II Mpox Associated with Intimate and Nonintimate Close Contact Among Men Who Have Sex with Men and Transgender Adults — United States, August 2022–July 2023. MMWR Morbidity and Mortality Weekly Report. 2024;73. doi:10.15585/mmwr.mm7340a2.
