# Supplementary material for: BharatSim: An agent-based modelling framework for India
Source: PLoS Comput Biol. 2024 Dec 30;20(12):e1012682. doi: 10.1371/journal.pcbi.1012682 (PMC11750085; doi:10.1371/journal.pcbi.1012682)
Supplement: Attachment — Submitted filename: BharatSim Review.pdf [file pcbi.1012682.s010.pdf]

Review of "BharatSim: An agent-based modelling framework for India" by Cherian et al., 2023.

The authors work to create a modular and customisable framework and codebase for running agent based model simulations - with a focus on applications in decision making for infectious disease epidemics. They focus on 3 modules for the same - a synthetic population generation engine, a simulation engine, and a visualisation engine.

The *synthetic population* module uses a blend of a Iterative Proportional Fitting (IPF) and a generative adversarial network - thus allowing them to use data from an older 2011 Indian census to glean geographical distribution across administrative units, while reinforcing the same with the Gridded Population of the World and the National Surveys conducted by the government for employment information and gender, age, weight, height distributions. The adversarial network approach works to create a robust population distribution - which is validated using machine learning regression models and various statistical metrics.

The *simulation engine* takes the synthetic population to the next logical step - by defining relationships, actions, and work and life schedules for the population. The authors work to classify adults, children, and toddlers with different relationships, classify them as home-bound or not, and assign schedules. They then assign means of commute and modes of transmission. The visualisation engine brings the modules together to allow for public consumption of the model's results.

The author's focus on creating a robust population simulator that has the potential to work in data poor environments (albeit with poorer results) is commendable, as most geographies with gaps in data collection require a starting point for decision making. The simulation engine's focus on ability to run counterfactual simulations is crucial - to allow simulating not just NPIs like curfews and lockdowns - but can in the future be used to simulate different scenarios wrt longer/shorter working weeks, and hybrid (remote/in-person) policies for work.

One small comment would be that, with the focus placed on testing the efficacy of NPIs, there could be a more detailed explanation on how other social gatherings of all kinds - ranging from restaurants and parks to movie theatres and festivals - aid transmission and how the effect of these gatherings was tested.

As the authors point out, these agent based models have the potential to be expanded to applications well beyond infectious disease epidemiology, ranging all the way to the social sciences and economics. The open source nature of the code allows maintenance and modification by the community at large, and is worth appreciating. To that end, it would be helpful if the raw datasets (or the exact sources thereof, when publicly available) are made available to the public - to allow easy recreation of the engines and results.

No major revisions are suggested at this time.
