# Supplementary material for: BharatSim: An agent-based modelling framework for India
Source: PLoS Comput Biol. 2024 Dec 30;20(12):e1012682. doi: 10.1371/journal.pcbi.1012682 (PMC11750085; doi:10.1371/journal.pcbi.1012682)
Supplement: Attachment — Submitted filename: BharatSim-responses-to-reviewers.pdf [file pcbi.1012682.s011.pdf]

Responses to Reviewers

Philip Cherian, Jayanta Kshirsagar, Bhavesh Neekhra, Gaurav Deshkar, Harshal Hayatnagarkar, Kshitij Kapoor, Chandrakant Kaski, Ganesh Kathar, Swapnil Khandekar, Saurabh Mookherjee, Praveen Ninawe, Riz Fernando Noronha, Pranjal Ranka, Vaibhhav Sinha, Tina Vinod, Chhaya Yadav, Debayan Gupta, Gautam I. Menon

March 28, 2024

We thank all three reviewers for their careful reading and comments. We have addressed all the points raised by the reviewers in the revised manuscript, as detailed point by point below. We have also made some related modifications and additions to the text. Our replies below, and all changes to the revised manuscript, are marked in blue.

Reviewer #1: I thank the authors for the opportunity to read their informative manuscript "BharatSim: An agent-based modelling framework for India". In this work, Cherian et al. develop a novel agent-based modeling (ABM) framework BharatSim. Crucially, this ABM contains pre-specified data and defaults for India, allowing for easier and more accurate scenario projections for this unique context. The ABM is also quite efficient and has extensive documentation and support. The scenarios developed and discussed in the manuscript include the impact of various NPIs and timing of school re-opening. These scenarios are relevant to development of public health planning and guidance; modern open-source computational tooling is quite a valuable step.

I found the software development described in this manuscript quite impressive and appreciated the thoughtful documentation. I can see substantial research value that will be derived from this work. In addition, the harmonization of the disparate Human Development Survey, Census, GPW population density, and others is quite valuable. Both are important contributions.

However, I have a number of methodological concerns that should be addressed. These include insufficient sensitivity analyses, unclear modeling assumptions, and conclusions that do not match the strength of the evidence. I have split my main concerns into a major and minor section below:

We thank the reviewer for these positive comments on our paper, in particular for their comments on the software development and documentation. We address their comments and questions in detail below.

Major

- It is my understanding that many of the datasets used in the manuscript are quite out of date and may not reflect the current Indian population. The clearest example of this may be the Census data, which I believe are from 2011 and likely do not accurately reflect India's fast-growing population to the resolution used in this manuscript. There are similar issues with the Gridded Population of the World, the Human Development Survey, and the IHDS-II datasets. Some form of statistical correction and/or additional supplementary analyses examining the sensitivity of results to out-of-date data would be justified. I believe this is a crucial issue because one of the main advances in this manuscript is combining these datasets and producing results uniquely appropriate for the Indian context.

We agree with the reviewer that this is an important concern. However, we note that most models directed at policy continue to be based on data from the 2011 census [1, 2], which is why we have chosen to use this data, as well as other survey-data, corresponding to this approximate point in time. This also potentially allows us to make comparisons to other modelling work which uses the same base data. However, updated data for some of the surveys from which we have drawn have been released since 2011. Additionally, the Niti Ayog, India's apex planning body, has provided population projections for India and its states, initialized from the 2011 census, across the period 2011-2036. However, this is still only at the level of entire states and not at the more granular level that we implement here.

We appreciate that modellers might wish to use a more updated population. We have therefore provide a second population, incorporating the projections from the Niti Ayog. This population is available from the BharatSim website (`bharatsim.ashoka.edu.in`) and a line describing its generation has been added at the end of Section 2.1.2 ("Population generation") in the revised manuscript (lines 231–241).

- I believe substantially more sensitivity analyses are needed. There are strong assumptions on the population distributions (uniform inside the population grids I believe?), the number of movements and distance moved between "home" and "work" patches, and the specification of the epidemiological scenarios. While I appreciate that these models are computationally intensive to run, it is impossible to evaluate the modeling framework without understanding which assumptions are robust and which change the results. There are certainly parameters to which the results are not robust — I believe those should be examined and reported.

We thank the reviewer for pointing this out. Agent-based models must, of necessity, incorporate a large number of assumptions. As described, we have checked that the statistical distribution of a number of synthetic population attributes are similar to those of the actual population. These results, where we have compared a generated synthetic population for the city of Mumbai with data drawn from the IHDS-II survey, have now been incorporated in Appendix S2 of the revised supplementary material for completeness.

We have made reasonable assumptions based on what is known about the population. Some of these assumptions are expected to be robust, especially those which are obtained from large population samples. However, other quantities – such as the average workplace occupancy, the distribution of travel distances, and the division of time between homes and workplaces – rely on specific assumptions for which no data currently exists. If such data were to become available in the future, it could be easily incorporated into the generation of our synthetic population.

As suggested by the referee, we have conducted further sensitivity analyses. We ran multiple simulations using populations in which (i) the distances between "home" and "work" patches are varied, (ii) the average workplace occupancy is varied, and (iii) the time spent at home is varied. These results have been incorporated in Appendix S4 of the revised supplementary material. We find that many of our numerical results change very little; the qualitative behaviour of our graphs remains the same, indicating that our results are robust against these assumptions. We stress that we cannot rule out whether our results are sensitive to non-intuitive distributions of these and other attributes of our synthetic population. However, these problems would be generic to any similar agent-based model.

- In all the epidemic scenarios, I am uncomfortable with the framing of the results. These results are not statistical fits to epidemic data and do not necessarily correspond to observed epidemic dynamics. These scenario-based models can project dynamics subject to particular assumptions. Greater emphasis on and transparency of the necessary assumptions would make

these results stronger and more believable. For example, I am unclear what key assumptions drive the finding in the school reopening example. It seems quite different than results in other settings (see e.g., `https://doi.org/10.1038/s41467-021-21249-6`). These deviations are not explained or contextualized and, to me, make the results less believable.

Since this paper is devoted to the description of the simulation framework and its potential uses for policy, we chose not to make the matching to observed data, with its associated problems of undercounting, varied interventions, delayed reporting and an evolving mix of variants, a central theme. Accounting, within these simulations, for the large fraction of undercounted infections and deaths in the real-world data for COVID-19 in India would have taken us away from the main thrust of this work.

However, we appreciate the reviewer's concerns. While we have not directly compared the simulation results from this paper to real-world data, our epidemic model is based on INDSCI-SIM, a 9-compartment model rigorously benchmarked to the first wave of COVID-19 in India [3]. The results of our, or indeed any, agent-based simulation can be made to agree with those of a differential-equation based "compartmental" model like that of INDSCI-SIM (Ref. [3]) by considering a well-mixed population in which all agents are in contact with each other, thereby removing any network structure. While each stochastic run of an agent-based simulation will necessarily give a different result, the ensemble average of these runs will reproduce the mean-field differential equation model. (For a discussion of this, see for example Ref. [4].) We have now added a subsection in the revised supplementary information where we have shown this explicitly by overlaying the results of our simulation with that of the associated compartmental model of INDSCI-SIM (see Appendix S3). Consequently, we could in principle modify our agent-based simulation to recreate all the results from Ref. [3].

The reviewer also draws our attention to the fact that our results differ from those in Di Domenico et al. [5], a modelling study that explores scenarios of partial, progressive, or full school reopening in France. In fact, this difference underlines the need for a synthetic population tailored to India for such investigations. We interpret our own results in the school-reopening example as showing the importance of hybrid immunity in curtailing disease spread, a modelling result of considerable importance in the debate around school-reopening in India. Some of the authors of this paper have found similar results in a different modelling study using a compartmental model with contact matrices specific to the Indian context [6]. Indeed, as we have shown in Fig 7 of the revised manuscript, at very low background seroprevalence, the reopening of schools did indeed cause a large rise in cases among children.

Our explanation for the difference between our results and those of Di Domenico et al. is as follows: the population structure and contact matrices in Di Domenico et al. differ considerably from our setting. Also, crucially, the fraction of agents in the 65+ category is over 21% in France, while it is less than as 7% in India. Given the strong dependence of the IFR on age, and the overall substantially larger median age of the French as opposed to the Indian population, we would expect mortality arising from school reopenings to impact the population far more substantially in France in comparison to India. An additional feature of the Indian IFR is its saturation for the elderly population, when compared to its continued exponential increase for the elderly in western populations. [3, 7].

We have added a paragraph reflecting this discussion in the main text in lines 767–774, citing the Di Domenico paper.

- I find the number of figures overwhelming. It would help if some of the model explanation and figures could move to the appendix to streamline the paper. I had trouble following which pieces were important for the model and which were ancillary choices.

We thank the reviewer for this helpful suggestion. We have moved some of the figures to the revised supplementary material. We hope that this improves the overall readability of the paper.

Minor

- I don't understand the framing in the Introduction of compartmental models vs. network-based models or ABMs (see: "Both network- and agent-based models must use many more assumptions than compartmental models require."). As I understand it, the model used in BharatSim is an extension of the SIR compartment model which generalizes the mixing of individuals within the population. This is a classic framing, but one I would still describe as compartment-based.

  We use the nomenclature of compartments only to label disease states of individuals, as this allows us to differentiate agents with different levels of infection (such as asymptomatic or severely infected) who might have different effects on the spread of the disease. We state this explicitly in line 463–464: "The disease states in these models can be labelled according to the compartments in associated compartmental models." References [8, 9, 10, 11, 12], amongst others, use a similar approach.

- I do not agree with the framing around "realism", well-mixed populations, and ODE approaches. These ABMs allow for extraordinarily detailed scenarios and investigation of complex dynamics, but they are fundamentally scenario projections. There is no fit to data and the results are not necessarily realistic. I don't see that as a flaw — on the contrary, it can be a real strength and allow us to examine unobserved possibilities.

  Compartmental models are one part of the space of ABMs. ABMs can be made to reproduce results of compartmental models, but can in addition also include social and other interaction network dynamics. Indeed, in our definitions, agent-based models contain compartmental models within them if each individual is assumed to interact with every other individual and residence times in each disease state are drawn from corresponding exponential distributions. Our use of the word realistic is meant to convey that such models generalize simple compartmental models in physically relevant ways to capture the complexity of human interactions.

  In principle, one could use BharatSim to simulate the progress of an epidemic, tuning a small subset of parameters so as to reproduce, for example, disease incidence or serosurvey results. However, we believe that the value of a study such as this is precisely the exploration of counterfactuals and the synergies of different NPIs, results that are more robust than those that would be obtained by direct comparison to, say, the observed number of cases or deaths in the COVID-19 example, both of which were known to be severely undercounted in countries like India. These are hard to convincingly study within compartmental models.

- Broader engagement with the literature for each of the scenarios would be appropriate. For example, there is a broad literature in variant dynamics (see e.g., `https://doi.org/10.1126/science.abj0113`, `https://doi.org/10.1098/rsif.2021.0900`, `https://doi.org/10.1126/science.abl9551`) and it would be useful to understand how the approaches used here fit in with this broader literature — with similar efforts for the other scenarios.

  Because our model can keep track of each agent's infection history, it is in principle easy for us to simulate any sequence of variants with given epidemiological characteristics. We describe a model scenario in Fig 12 of the revised manuscript which shows the consequence of an infection by a more transmissible variant following an initial infection by a less transmissible one. (The situation in India after the first wave was more complicated, since multiple variants, including Kappa, were present in the population before the emergence of the Delta variant,

and each sub-region of India had its unique profile of coexisting variants.) Given estimates for immune escape including reinfections and breakthrough infections, we can *post-facto* generate the resulting epidemic curves, as well as scope out the potential consequences of any number of different variants of specified transmissibility and with different severity of disease. We leave the detailed study of these effects to future work.

Given the importance of variant dynamics for COVID-19, we have added some text reflecting the discussion above in the revised manuscript (lines 803–805), also including references to the works cited above.

- I would appreciate if the supplement could include definitions and model values associated with each parameter in the supplement "S3: Description of the compartmental model"

  We thank the reviewer for pointing this out – this was an omission on our part which is now rectified. We have included a table with the parameter definitions in this supplement. However, it should be noted that the differential equations in "Description of the compartmental model" describe a well-mixed scenario in which the transitions between compartments are Markov processes. Consequently, the residence times are exponentially distributed. In the agent-based model described in the main paper, however, we have extended this to incorporate lognormally-distributed residence times, for which no equivalent simple description exists in conventional ODE-based compartmental models.

- I would appreciate additional supplementary figures and analysis of the joint distribution between population parameters. Which parameters are correlated? Is it the case that the parameters we expect to be uncorrelated are actually uncorrelated?

  During the generation of the synthetic population, we did, in fact, examine the joint-distributions of weight, height, age, and gender, comparing these to known survey data. The supplementary material now includes a discussion of the correlations between a number of attributes in the synthetic and survey data. The agreement was found to be very good. However, we note that of these attributes, only age is relevant in our disease modeling simulations, since it is central to the agent-dependent disease progression. We cannot rule out, of course, the absence of subtle correlations in our model population vis-à-vis the true data, only pointing out that we could also have simulated our model with the exact population if this information were available.

Overall, I found the modeling software and data harmonization described in this work to be a valuable contribution. I appreciated the excellent software development and thoughtful model development described in the manuscript.

We thank the reviewer for these positive remarks and appreciate the thought that went into them.

Reviewer #2:

- In the introduction a proper survey of agent-based models used for studying other infectious diseases should be included.

  We thank the referee for this suggestion. In addition to the models described in lines 840–850, we have included a number of references to agent-based models and their applications in the revised manuscript (lines 51–53).

- In Section 2.1.2, how are capturing simple attributes like height, weight, age and sex relevant to the study of Covid-19. This should be supported using references. Why not include attributes like blood group, nutrition etc. which may be more relevant.

The aim of this section was to describe the creation of a synthetic population that would have broader uses than the study of infectious diseases alone. For this reason, we have benchmarked in some detail the demographic parameters of the population. We note that in the full synthetic population described here, each agent has 44 attributes, including multiple co-morbidities, all of which are obtained from survey datasets. We appreciate that this was not made clear in the original paper and have now made this explicit in lines 486–489. More information on the different attributes of the synthetic population can now be found in Appendix S2.

The sample datasets that are used to generate our population include no information on individual agents' blood groups or nutrition. While the synthetic population includes information on gender, weight and height, among other attributes, none of these are used in our models of disease spread.

- There is no validation of the contact patterns, workplaces, travel using source/survey data. Only validation for simple attributes have been included. Authors should also validate the other assumptions in the synthetic populations.

We understand the reviewer's concern. We have addressed this in our response to a related question from Reviewer 1, which we reproduce here:

We thank the reviewer for pointing this out. Agent-based models must, of necessity, incorporate a large number of assumptions. As described, we have checked that the statistical distribution of a number of synthetic population attributes are similar to those of the actual population. These results, where we have compared a generated synthetic population for the city of Mumbai with data drawn from the IHDS-II survey, have now been incorporated in Appendix S2 of the revised supplementary material for completeness.

We have made reasonable assumptions based on what is known about the population. Some of these assumptions are expected to be robust, especially those which are obtained from large population samples. However, other quantities – such as the average workplace occupancy, the distribution of travel distances, and the division of time between homes and workplaces – rely on specific assumptions for which no data currently exists. If such data were to become available in the future, it could be easily incorporated into the generation of our synthetic population.

As suggested by the referee, we have conducted further sensitivity analyses. We ran multiple simulations using populations in which (i) the distances between "home" and "work" patches are varied, (ii) the average workplace occupancy is varied, and (iii) the time spent at home is varied. These results have been incorporated in Appendix S4 of the revised supplementary material. We find that many of our numerical results change very little; the qualitative behaviour of our graphs remains the same, indicating that our results are robust against these assumptions. We stress that we cannot rule out whether our results are sensitive to non-intuitive distributions of these and other attributes of our synthetic population. However, these problems would be generic to any similar agent-based model.

- What are these numbers in Table 2? Are these correlations?

We thank the referee for pointing this out and it was indeed unclear in our original draft. The numbers mentioned in the table are the model's predictive accuracy when trained and tested on real population data, as compared to when an identical model is trained on synthetic population data and tested on real population data.

We have included this information in the table's heading and caption.

- The evaluation of the model is based only on simulations. Why not use real data to verify the predicted infection trajectories wrt the interventions? The authors should include a comparison based on real data.

  We understand the reviewer's concern. We believe we have addressed this in our response to a related question from Reviewer #1. We reproduce our reply here:

  Since this paper is devoted to the description of the simulation framework and its potential uses for policy, we chose not to make the matching to observed data, with its associated problems of undercounting, varied interventions, delayed reporting and an evolving mix of variants, a central theme. Accounting, within these simulations, for the large fraction of undercounted infections and deaths in the real-world data for COVID-19 in India would have taken us away from the main thrust of this work.

  However, we appreciate the reviewer's concerns. While we have not directly compared the simulation results from this paper to real-world data, our epidemic model is based on INDSCI-SIM, a 9-compartment model rigorously benchmarked to the first wave of COVID-19 in India [3]. The results of our, or indeed any, agent-based simulation can be made to agree with those of a differential-equation based "compartmental" model like that of INDSCI-SIM by considering a well-mixed population in which all agents are in contact with each other, thereby removing any network structure. While each stochastic run of an agent-based simulation will necessarily give a different result, the ensemble average of these runs will reproduce the mean-field differential equation model. (For a discussion of this, see for example Ref. [4].) We have now added a subsection in the revised supplementary information where we have shown this explicitly by overlaying the results of our simulation with that of the associated compartmental model of INDSCI-SIM (see Appendix S3). Consequently, we could in principle modify our agent-based simulation to recreate all the results from Ref. [3].

- On page 26, the authors claim that the model can be applied to other infectious diseases like typhoid. I would suggest that the authors include such an example based on typhoid. This would make the manuscript more applicable as studying Covid-19 at this point does not have much applicability.

  Given that the focus of this paper was to introduce BharatSim as a new agent-based modelling framework and explore the sort of questions that it can address, for simplicity we chose to confine ourselves in this paper to a detailed exploration of COVID-19 as a model of a respiratory disease in the mould of WHO's Disease X. Exploring other infectious diseases would have resulted in a considerable increase in the length of this paper. We choose, therefore, to leave such explorations to later work. However, how we could extend BharatSim to include diseases that spread via environmental reservoirs is of considerable interest to us.

Reviewer #3:

The authors work to create a modular and customisable framework and codebase for running agent based model simulations - with a focus on applications in decision making for infectious disease epidemics. They focus on 3 modules for the same - a synthetic population generation engine, a simulation engine, and a visualisation engine.

The *synthetic population* module uses a blend of a Iterative Proportional Fitting (IPF) and a generative adversarial network - thus allowing them to use data from an older 2011 Indian census to glean geographical distribution across administrative units, while reinforcing the same with the Gridded Population of the World and the National Surveys conducted by the government for employment information and gender, age, weight, height distributions. The adversarial network approach works to create a robust population distribution - which is validated using machine

learning regression models and various statistical metrics.

The *simulation engine* takes the synthetic population to the next logical step - by defining relationships, actions, and work and life schedules for the population. The authors work to classify adults, children, and toddlers with different relationships, classify them as home-bound or not, and assign schedules. They then assign means of commute and modes of transmission. The visualisation engine brings the modules together to allow for public consumption of the model's results. The author's focus on creating a robust population simulator that has the potential to work in data poor environments (albeit with poorer results) is commendable, as most geographies with gaps in data collection require a starting point for decision making. The simulation engine's focus on ability to run counterfactual simulations is crucial - to allow simulating not just NPIs like curfews and lockdowns - but can in the future be used to simulate different scenarios wrt longer/shorter working weeks, and hybrid (remote/in-person) policies for work.

One small comment would be that, with the focus placed on testing the efficacy of NPIs, there could be a more detailed explanation on how other social gatherings of all kinds - ranging from restaurants and parks to movie theatres and festivals - aid transmission and how the effect of these gatherings was tested. As the authors point out, these agent based models have the potential to be expanded to applications well beyond infectious disease epidemiology, ranging all the way to the social sciences and economics. The open source nature of the code allows maintenance and modification by the community at large, and is worth appreciating. To that end, it would be helpful if the raw datasets (or the exact sources thereof, when publicly available) are made available to the public - to allow easy recreation of the engines and results.

No major revisions are suggested at this time.

We thank the reviewer for their very positive comments.

The raw datasets from which the synthetic population is generated are now available online on the BharatSim website (`bharatsim.ashoka.edu.in`) and GitHub page (`www.github.com/bharatsim`). We have included a line about this in the revised manuscript (lines 227–230).

We have studied how seasonal variations in contact patterns arising out of social gatherings affect the transmission of COVID-19, using contact matrices obtained from real-world data, in BharatSim. This work has appeared in Ref. [13] and is part of a broader investigation into the seasonality of influenza in northern India. This, too, has now been added to the manuscript (lines 834–839).

References

[1] SPRF T. New India's Policies from Old India's Data: The 16th Census Delay; 2023. Available from: https://sprf.in/new-indias-policies-from-old-indias-data-the-16th-census-delay-and-its-impact/.

[2] Sivapriyan ETB. In the dark without data: Census delay leaves citizens in limbo; 2024. Available from: https://www.deccanherald.com/india/in-the-dark-without-data-census-delay-leaves-citizens-in-limbo-2878741.

[3] Hazra DK, Pujari BS, Shekatkar SM, Mozaffer F, Sinha S, Guttal V, et al. Modelling the first wave of COVID-19 in India. PLOS Computational Biology. 2022;18(10):e1010632. doi:10.1371/journal.pcbi.1010632.

[4] Rahmandad H, Sterman J. Heterogeneity and Network Structure in the Dynamics of Diffusion: Comparing Agent-Based and Differential Equation Models. Management Science. 2008;54(5):998–1014. doi:10.1287/mnsc.1070.0787.

[5] Di Domenico L, Pullano G, Sabbatini CE, Boëlle PY, Colizza V. Modelling safe protocols for reopening schools during the COVID-19 pandemic in France. Nature Communications. 2021;12(1):1073. doi:10.1038/s41467-021-21249-6.

[6] Mozaffer F, Cherian P, Krishna S, Wahl B, Menon GI. Effect of hybrid immunity, school reopening, and the Omicron variant on the trajectory of the COVID-19 epidemic in India: a modelling study. The Lancet Regional Health - Southeast Asia. 2023;8:100095. doi:10.1016/j.lansea.2022.100095.

[7] Laxminarayan R, Wahl B, Dudala SR, Gopal K, Mohan B C, Neelima S, et al. Epidemiology and transmission dynamics of COVID-19 in two Indian states. Science (New York, NY). 2020;370(6517):691–697. doi:10.1126/science.abd7672.

[8] Narassima MS, Anbuudayasankar SP, Jammy GR, Sankarshana A, Pant R, Choudhury L, et al. An agent-based model to assess coronavirus disease 19 spread and health systems burden. International Journal of Electrical and Computer Engineering (IJECE). 2022;12(4):4118–4128. doi:10.11591/ijece.v12i4.pp4118-4128.

[9] Kerr CC, Stuart RM, Mistry D, Abeysuriya RG, Rosenfeld K, Hart GR, et al. Covasim: An agent-based model of COVID-19 dynamics and interventions. PLOS Computational Biology. 2021;17(7):e1009149. doi:10.1371/journal.pcbi.1009149.

[10] Agrawal S, Bhandari S, Bhattacharjee A, Deo A, Dixit NM, Harsha P, et al. City-Scale Agent-Based Simulators for the Study of Non-pharmaceutical Interventions in the Context of the COVID-19 Epidemic. Journal of the Indian Institute of Science. 2020;100(4):809–847. doi:10.1007/s41745-020-00211-3.

[11] Adiga A, Chu S, Eubank S, Kuhlman CJ, Lewis B, Marathe A, et al. Disparities in spread and control of influenza in slums of Delhi: findings from an agent-based modelling study. BMJ Open. 2018;8(1):e017353. doi:10.1136/bmjopen-2017-017353.

[12] Perez L, Dragicevic S. An agent-based approach for modeling dynamics of contagious disease spread. International Journal of Health Geographics. 2009;8(1):50. doi:10.1186/1476-072X-8-50.

[13] Nagpal S, Kumar R, Noronha RF, Kumar S, Gupta D, Amarchand R, et al. Seasonal variations in social contact patterns in a rural population in north India: Implications for pandemic control. PLOS ONE. 2024;19(2):e0296483. doi:10.1371/journal.pone.0296483.
