# Supplementary material for: BharatSim: An agent-based modelling framework for India
Source: PLoS Comput Biol. 2024 Dec 30;20(12):e1012682. doi: 10.1371/journal.pcbi.1012682 (PMC11750085; doi:10.1371/journal.pcbi.1012682)
Supplement: Attachment — Submitted filename: BharatSim-responses-to-reviewers.pdf [file pcbi.1012682.s012.pdf]

Responses to Reviewers

Philip Cherian, Jayanta Kshirsagar, Bhavesh Neekhra, Gaurav Deshkar, Harshal Hayatnagarkar, Kshitij Kapoor, Chandrakant Kaski, Ganesh Kathar, Swapnil Khandekar, Saurabh Mookherjee, Praveen Ninawe, Riz Fernando Noronha, Pranjal Ranka, Vaibhhav Sinha, Tina Vinod, Chhaya Yadav, Debayan Gupta, Gautam I. Menon

October 13, 2024

We thank the editor and reviewers for re-evaluating our manuscript.

We note that the comments of reviewers have overall been very positive regarding the manuscript. In the previous review, reviewer #1 found the software development and documentation impressive, in additional to recognising the importance of the harmonisation of the IHDS, census, and gridded population density data. Reviewer #3 had no major revisions to recommend. The other reviewers had specific broad concerns, some of which were reiterated by the editor.

In the points below, we address the few remaining concerns of the reviewers. Our replies are colored in blue, and quotes from the previous referee report are coloured in red and *italicised*. The referenced line numbers correspond to the lines in the revised document

Reviewer #2:

- *The evaluation of the model is based only on simulations. Why not use real data to verify the predicted infection trajectories wrt the interventions? The authors should include a comparison based on real data.*

  This comment has not been addressed. The authors need to give an assessment of their results with some real world data.

  We were initially reluctant to add more text to an already very long paper (a point made by other reviewers) whose primary focus was methodological. However, we do appreciate that the value of the paper would be substantially increased if we illustrated a real-life application and thank both reviewer and editor for emphasizing this point.

  We now include an additional section in the Supplementary Information (Appendix S8) in which we discuss the use of BharatSim in epidemic forecasting using data from the first wave of COVID-19 in Pune. We additionally include the following paragraph in lines 730–749 of the revised manuscript that provides context:

  We recognise the utility of at least an approximate comparison to real data. We have chosen to use our model to describe the onset of the COVID-19 pandemic in the city of Pune, comparing model results to real data. We choose a time period between the 1st of March and the 15th of July, 2020 for this comparison for the following reasons: (i) the simulations are initiated when the lockdown started so spread would primarily have been driven by in-house transmission, (ii) the virus dynamics was largely dominated by a single variant, and (iii) we can study the impact of the relaxation of the nation-wide lockdown on cases.

  This direct comparison is shown in Appendix S8. Briefly, our results are consistent with (i) a relaxation of the first lockdown leading to an increase in cases, (ii) a large background of

undetected infection, arising mainly from asymptomatic cases, (iii) an indication that the epidemic arising from the initial strain had run its course by the end of the second lockdown and that (iv) that a complex mix of more transmissible variants would have determined the later-time course of the disease. These agree with a retrospective analysis [59, 72].

- *The model should be extended to include one other infectious disease to show applicability to modelling infectious diseases other than covid.*

  This comment was made earlier also but not addressed.

  Here too we take the reviewers point and, to address this, we have now included a detailed study of a different infectious disease (mpox) in Appendix S9.

  A global outbreak of mpox began in May 2022 and continues to this day, with the WHO declaring it a Public Health Emergency of International Concern (PHEIC) in July, 2022. Additionally, a new strain of the virus (Clade Ib) has begun to spread to countries that had not previously this disease. We use BharatSim to address how the disease can spread through sexual and non-sexual contact networks, and further look at how the introduction of transmission to work networks can lead to a situation of prolonged prevalence in the population.

  In addition to a paragraph in the Introduction of the main paper (lines 105–112), we have also included an explanation of our results in the Discussion (lines 849–861) which we reproduce here:

  To further demonstrate the versatility of BharatSim beyond this specific application, we have used it to model the spread of mpox in a population of men who have sex with men (MSMs) and their associate sexual and non-sexual networks. To our knowledge, only two agent-based models of mpox are present in the literature [79, 80].) Our results, described in Appendix S9 are the following: if contact is solely via MSM networks, we see a fast spread across this network that rises and falls over a relatively fast time-scale. If, in addition, we assign a low probability of transmission between agents in the household of infected MSMs, we see a slower rise and fall in cases that also peaks at a later time. The nature of the spread depends upon the level of the initial infection in the non-MSM population. The interaction of the MSM and non-MSM population can lead to an unusual two-peak structure in the numbers of infected with time when the infection is initially dominated by non-MSM agents. Expanding the possibility of such transmission to work networks can lead to a situation of prolonged prevalence.

Reviewer #4: Cherian et al. present a new software package BharatSim, which is an agent-based modeling framework tailored to India. This ABM is applied to a number of different scenarios, demonstrating its versatile parameterization and the wide range of provided data sources.

Despite the impressive software engineering described in the manuscript, I remain uncomfortable with the presentation of the results and unconvinced by the underlying dataset. Although the additional sensitivity analyses are helpful, I do not believe the ameliorate the concerns around the accuracy of the data for specific policy recommendations. I elaborate on my two main concerns below:

- The modeling framework is geared to answer policy questions and describe dynamics in a named Indian city. The findings are used to make specific policy recommendations and quite generic usage is advocated in the Discussion. I am not convinced that this model can be used to answer these specific, detailed policy questions without specific, detailed data and numerical fitting to that data. Rather, this model can be used to examine generic questions subject to specific modeling assumptions and the results would not necessarily be

applicable to any particular real-world context. I believe the results and particularly the Discussion need substantial additional edits to change the level of specificity in the framing and recommendations.

We first refer the referee and editor to our reply to Reviewer 2, where we show that BharatSim can be used with real data as input to project the course of infections, with the real-life example of the early part of the first wave in India. Data on cases can be used to extract preliminary estimates for the reproductive ratio, using well-defined and benchmarked statistical methods (a Bayesian approach developed by Bettencourt and Ribeiro (2008), later modified by Systrom (2021)), which maps on to associated quantities in our agent-based model. These can be further refined as more data is obtained. We note that our model accounts for two major interventions – the lockdowns that bracketed a period where the city was opened up – that were imposed.

As can be seen from the fits displayed, the model does reasonably well in projecting the initial course of the disease vis-à-vis. actual data. This exercise could actually have been done in real time based on the limited data available at the onset of the first wave in India. We hope this example suffices to support our commitment to the use of "specific, detailed data and numerical fitting to that data".

We also refer the referee to the additional work included in the resubmitted manuscript regarding mpox modeling. Here we draw from published data regarding MSM networks across three Indian cities and known epidemiological parameters for mpox, to explore a number of scenarios for mpox spread, currently an issue under discussion in Indian public health circles.

Finally, as a simulation framework, BharatSim's goal is to create a space within which modellers can construct their own disease progressions and address specific questions with a synthetic population tailored to the Indian context. In order to illustrate this, we provided three examples of the sorts of questions that modellers might study with the framework. However, we stress again that none of the numbers, or indeed, the basic disease progression, are hard-coded into the framework. Indeed, as we have shown in the newly added Appendix S9, the BharatSim framework can quite easily be used to model the spread of mpox, a disease that is predominantly sexually transmitted. We have used a different disease progression (an SEIR model), as well as a different contact network structure to distinguish between sexual and non-sexual contacts. We modeled the actual network of MSM contacts in an Indian city, given the prior literature that studied such networks, and known values for the ranges of parameters characterizing mpox spread.

Our purpose here, as we have pointed out in the main text (lines 745–749) is not intended to precisely reproduce the different complexities of modelling diseases or even provide a detailed road-map to policy recommendations, but rather to describe a framework within which such questions can even be addressed. We believe that the way we have presented the data and results make only this claim and no more.

- The data are not specific and detailed to the current context. In general, older datasets are useful and provide value, but not when answering specific, detailed modeling questions on recent disease dynamics. I agree that it is appropriate to use these datasets as the best available data, but I still do not understand which modeling assumptions are influential. This question was not answered by the additional sensitivity analyses. In particular, I have trouble figuring out what changes in an updated dataset would have in this modeling framework. Is it anything? Why or why not? How do these uncertainties change the certainty around policy recommendations?

  We understand the reviewer's concern. To clarify, we note that while we have described

a technique to generate a synthetic population with a large number of attributes, at the individual-level, the age distribution is the only relevant parameter in our simulations, since it determines the agent-dependent disease progression. Other parameters are part of the synthetic population but are not required explicitly in the simulations we carry out.

We believe the age-distributions to be robust since they are obtained from several different sources. To make this point more specifically, from the Niti Aayog's population projections (which we have used to create a projected population for 2021 as we understand that some modellers might wish to use a more updated population), the distribution of ages changes only marginally at the state level between the 2011 census and the 2021 statistical projection. For this reason, we do not believe that even if we had more up-to-date micro-level data – which is unavailable anyway – it would not have changed any of our conclusions.

At the network level, the density and time-spent at different locations is another factor that strongly influences disease spread and the effectiveness of interventions. However, as we have shown in the sensitivity analyses of Appendix S4, variations of both these numbers within reasonable values lead to qualitatively identical results. Consequently, we believe our results to be robust against such changes.

Although the software engineering and data collation described in this manuscript are valuable, I remain unconvinced by the presentation of results. This concern is further exacerbated by the age of the underlying data for the specific policy recommendations presented.

We have done the most possible with existing data sources – the problem, simply stated, is that India has not conducted a country-wide census since 2011. Additionally, we stress that the quality and depth of data is a problem with virtually every LMIC. To impose too high a standard here, standards more appropriate to HICs with vastly more micro-level data availability, would ensure that no modeling work would ever be published from an LMIC that had to rely on any official data source, especially from India which our model focuses on.

In India a census is by now several years overdue. *Every* modeling exercise has had to make do with the older census data or, at a stretch, the broad increases at the state level that government think-tanks have made available. (These are themselves obtained from statistical projections and not from ground-level data collection.) As the quality of data improves, with the next Indian census, updated data can immediately be included in the updated synthetic population. But for now, we stress that our modeling work is consistent with, or improves upon, all other similar efforts in the Indian context.

Finally, we stress that in contrast to the term "specific policy recommendations" that the reviewer highlights, we make no such specific recommendations. We merely illustrate how BharatSim can be used – in principle – to address such questions. We understand that users who wish to make any specific recommendations will be guided appropriately by our caveats regarding data quality. They are free to substitute their own synthetic populations of choice if they wished to do so, using only the versatility of the simulation engine described here to do their calculations.
